# Discovering search behaviour in black garden ant trajectories

**DOI:** 10.1371/journal.pone.0327957

**Published:** 2026-07-29

**Authors:** Perrine Bonavita, Marius Albino, Jacques Gautrais, Vincent Fourcassié, Maud Combe, Loic Lacour, Simon Eibner, Christian Jost

**Affiliations:** 1 Centre de Recherches sur la Cognition Animale (CRCA), Centre de Biologie Intégrative (CBI), CNRS, Université de Toulouse, Toulouse, France; 2 Department of Energy and Process Engineering, Centre RAPSODEE, IMT Mines Albi, Albi, Occitanie, France; 3 Laboratoire Plasma et Conversion d’Energie (LAPLACE), CNRS, Université de Toulouse, Toulouse, France; Universidade de São paulo, BRAZIL

## Abstract

Exploratory behaviour plays an important role in many animals, in particular for social insects who have to feed and protect a whole colony. In a laboratory study, Khuong *et al* (2013) studied how workers of the black garden ant *Lasius niger* move around in an unknown environment. They assumed that, in a homogeneous arena with no visual cues, ants had no information about their position in space. Based on this hypothesis, they modelled ant movement in a Boltzmann Walker framework which describes an ant’s random walk as a series of straight segments separated by reorientation events. They found that, on a plain horizontal surface, an ant’s heading does not influence its speed, segment length and reorientation decision, thus leading to diffusive trajectories. However, published experiments indicate that *L. niger* ants are not completely devoid of directional information, even in standard laboratory setups with no obvious visual landmarks. Moreover, many ant species are known to develop specific search strategies when they want to find a particular place in space, a situation that may apply to the data Khuong *el al* analysed. We re-analysed their data on a plain horizontal surface, this time taking into account the ant’s heading in relation to its starting point in the arena. We discovered that the distributions of segment lengths and reorientation angles are modulated by the ant’s orientation in relation to its starting point. By simulating these biased trajectories, we show that this modulation leads to an area-restricted search behaviour. We also show that this modulation considerably accelerates the return times of ants to their starting point when they find themselves far from it. We conclude that not taking into account the animal’s cognitive abilities in data analysis may lead to incomplete or biased conclusions. The discovered search behaviour in *L. niger* can play a significant role in the colony’s exploration and foraging ecology.

## Introduction

The study of movement in animals asks several fundamental questions, notably why, how, when and where animals move [1,2]. Among the different types of movement displayed by animals, exploratory behavior (also called range search) and search behavior (also called area-restricted search) have received a lot of attention in the literature. Exploratory behavior is performed by animals when they need to locate a resource, such as a food source, a mate or a shelter at an unknown location, while search behavior is performed when they need to locate a resource at a known location, such as a nest or a food source that has not been depleted. Exploratory behavior has been mostly studied in animals that move on large distances and that do not have a stable place to rest, while search behavior has been mostly investigated in central place foragers which must return to their nest or shelter at the end of each foraging trip. For example, search behavior has been extensively studied in ants, which usually occupy a durable nest and are known to exploit stable food resources such as aphid colonies [3–7]. Therefore, in the context of exploratory or search behavior, the question “why” focuses on the reasons why the animal moves, while the question “how” explores the movement strategies it uses (e.g., random walks [4,8,9], Lévy walks [10], path integration [11,12], or others).

Here, we will address the ’how’ question in the case of the movement of the black garden ant *Lasius niger* when passively transported and released on an unknown area. Earlier work performed by Khuong *et al* [13] suggests that in this situation, these ants move according to a correlated random walk [14] (CRW), which results in a diffusion type exploration [15,16]. However, while this movement strategy is reasonably efficient for exploratory movement [9,17], it is not adapted to perform search behavior, i.e., to quickly find a specific location and come back to it. In absence of pheromone trails, *L. niger* is known to be able to navigate in its environment through the use of memorized visual landmarks [18–21] or home range marking [22]. Therefore, in the situation in which Khuong *et al* [13] operated, in which ants are passively transported by the experimenter, one would expect that ants should perform search behavior around their release point, in order to regain the familiar landmarks around the place near the nest where they have been collected [23]. It is therefore surprising that they should do simple correlated random walks, without any attempt to search for the point where they have been captured.

In this paper we will re-analyse Khuong *et al*’s data by changing the way the trajectories are analysed. We use their modeling framework, the Boltzmann walker or correlated random walk. However, instead of analyzing the trajectories within an arbitrary external reference frame, we analyse them within an egocentric reference frame, with respect to the direction pointing to the release point of the ants on the novel area. This allows us to show that the Boltzmann walker characteristics depend on the idiothetic information pointing towards the location of the release point of the ants. By using non-parametric trajectory simulations, we then compare Khuong *et al*’s and our modelling approach and assess how the two approaches affect the exploration efficiency of ants and their spatio-temporal distribution with respect to their release point. To investigate whether visual cues are involved in the observed search behavior, we then compare the trajectories of ants tested in experiments run either under white or red light. Since *L. niger* is assumed not to be able to perceive red light, we expected that ants should not perform a typical search behavior under red light. We end by a discussion on the link between the modified Boltzmann walker model we used and macroscopic diffusion type equations.

## Materials and methods

Trajectories on flat horizontal surfaces were downloaded from the ESM of Khuong *et al.* [13] (Dataset S1 [24]). The ant species studied and the experimental procedures are described in their paper. There are 69 trajectories. Note that ants were transported on a paint brush from their nest directly to the experimental area (a canvas free of any colony odour), which is the reason why we think that ants will immediately be in a search behavior mode.

**Fig 1:**
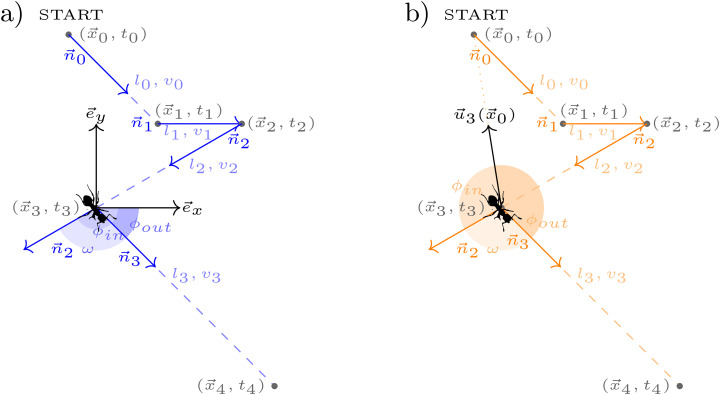
Example of the first four positions of an ant trajectory represented in the Boltzmann walker framework as a series of straight segments of length $l_{i}$ (dotted lines along which the ant moves with speed $v_{i}$ in direction ${\vec{n}}_{i}$) with turning angles $\omega_{i}$ between two successive segments. Ant orientation can be either characterised with respect to the ${\vec{e}}_{x}$ direction (*x*-axis), method used in Khuong *et al*’s (2013) work ($\Phi_{x}$ approach in blue (a)), or with respect to the direction ${\vec{u}}_{i}({\vec{x}}_{0})$ towards the release point of the ant ($\Phi_{\vec{u}}$ approach in orange (b)). If the ant is currently at position $\left({\vec{x}}_{i},t_{i}\right)$ then $\phi_{in,x,i}$ or $\phi_{in,\vec{u},i}$ refer to the orientation of the incoming segment and $\phi_{out,x,i}$ or $\phi_{out,\vec{u},i}$ to that of the outgoing segment. ${\vec{n}}_{i}$ is the unit vector in direction of segment *i* with length $l_{i}$.

According to the Boltzmann walker theoretical framework, a trajectory is modelled as a series of straight segments (also called free path) of variable length *l* (along which the ants move with speed *v*) with turning angles ω between them (see Fig 1). The statistical description of the segment length, associated speed and turning angle distributions, permits to characterise the trajectories. In particular, it allows to assess how these distributions change as a function of ant orientation (or heading) ϕ, be it arbitrary as in Khuong *et al* (they assessed ant orientation with respect to the arbitrary *x*-axis of the filming camera, we therefore term this the $\Phi_{x}$ approach), or with respect to the direction $\vec{u}$ pointing back to the starting point of the ant trajectory (therefore termed the $\Phi_{\vec{u}}$ approach). See Fig 1 for these two approaches and the respective ant orientations. A trajectory is analysed with the following model in mind: behavioral decisions occur at each turning point between two successive segments, with $v_{i}\sim v(\phi_{out})$, $l_{i}\sim l(\phi_{out})$ and $\omega_{i}\sim \omega (\phi_{in})$ (∼ means “drawn in a certain probability density function”, $\phi_{in}$ and $\phi_{out}$ refer to the orientation of the incoming and outgoing segments assessed at the decision point, which depend on the chosen approach, see Fig 1).

The original trajectories provided in Khuong *et al* ESM (Dataset S1 [24]) were sampled at 25 fps. To apply the Boltzmann walker analysis, we adopted the same segmentation algorithm as described in [13]. This segmentation aims at grouping together the positions that belong to the same segment *l*. The algorithm works as follows: initially, the orthogonal distance from each point ${\vec{x}}_{i}$ to the segment [${\vec{x}}_{i-1},{\vec{x}}_{i+1}$] is computed for all points. Then, the point with the smallest distance is removed from the trajectory. The orthogonal distances of the neighboring points of the removed point are then updated, and the process is repeated until the smallest distance found exceeds the threshold ε. We used the same ε as in [13] (their Fig S2 in S1 File), ε=1.7 mm. The authors had found through a simulation analysis that this value minimises biases in parameter estimation [25]. The segmented trajectory thus consists of a subset of the original points and the ant’s timestamps at each point remain unchanged.

The segmented trajectories can now be characterised by the distributions of the three variables used in the Boltzmann walker, *i.e.,* segment length *l*, speed *v* and turning angle ω (Fig 1). For each point of the segmented trajectory ${\vec{x}}_{i}$ at a given time $t_{i}$ (except the start and end points), we compute these three quantities, together with the heading angles $\phi_{in}$ and $\phi_{out}$ with respect to the $\Phi_{x}$ or the $\Phi_{\vec{u}}$ approach (see above and Fig 1). It is convenient to use the walk direction unit vector ${\vec{n}}_{i}$. Then, by definition of the dot product and the cross-product, we get ${\vec{n}}_{i-1}\cdot {\vec{e}}_{x}=\cos(\phi_{in,x,i})$, $\left||{\vec{n}}_{i-1}\times {\vec{e}}_{x}||=\sin(\phi_{in,x,i}\right)$, ${\vec{n}}_{i}\cdot {\vec{e}}_{x}=\cos(\phi_{out,x,i})$ and $\left||{\vec{n}}_{i}\times {\vec{e}}_{x}||=\sin(\phi_{out,x,i}\right)$ for the $\Phi_{x}$ approach (Fig 1.a). For the $\Phi_{\vec{u}}$ approach (Fig 1.b), these angles depend on the unit vector ${\vec{u}}_{i}({\vec{x}}_{0})$ pointing towards the starting point of the trajectory: ${\vec{n}}_{i-1}\cdot {\vec{u}}_{i}({\vec{x}}_{0})=\cos(\phi_{in,\vec{u},i})$, $\left||{\vec{n}}_{i-1}\times {\vec{u}}_{i}({\vec{x}}_{0})||=\sin(\phi_{in,\vec{u},i}\right)$, ${\vec{n}}_{i}\cdot {\vec{u}}_{i}({\vec{x}}_{0})=\cos(\phi_{out,\vec{u},i})$ and $\left||{\vec{n}}_{i}\times {\vec{u}}_{i}({\vec{x}}_{0})||=\sin(\phi_{out,\vec{u},i}\right)$. $\phi_{in}$ and $\phi_{out}$ are then computed with the atan2 function (atan2(x,y) is the polar angle of the point (x,y)≠(0,0) taking values in [−π,π]). After discretising the interval [−π,π] into 8 angular sectors of the same size with mean values 0, π/4, π/2, ..., we assign each value of *l*, *v*, and ω to the corresponding sector according to the value of ϕ ($\phi_{in}$ for ω, $\phi_{out}$ for *l* and *v*) for each approach. For example, all values of *l* with a $\phi_{out}\in (-\pi /8,\pi /8)$ are assigned to the sector with mean value 0. The discretisation of heading angles into this finite number of angular sectors is primarily motivated by statistical robustness and facilitates direct comparison with previous studies using the same framework [25].

Then, for each trajectory and for each sector, we characterised the Boltzmann walker variables with the following parameters:

### Turning angle distribution (also called phase function)

The forward persistence and the left-right asymmetry of the distributions of the turning angles ω (see Figs S1.1, S1.2, S1.7 and S1.8 in the S1 File) are characterised by the mean cosine $g_{\omega }$ and mean sine $s_{\omega }$ [9], respectively. They are estimated by the arithmetic mean of $\cos(\omega_{i})$ ($={\vec{n}}_{i-1}\cdot {\vec{n}}_{i}$) values and the arithmetic mean of $\sin(\omega_{i})$ ($=||{\vec{n}}_{i-1}\times {\vec{n}}_{i}||$) values, respectively [26]. The values of both the $g_{\omega }$ and $s_{\omega }$ statistics lie in the interval [−1,1]. The mean cosine is a measure of the strength of the persistence in the direction of motion. Values close to $g_{\omega }\approx 1$ correspond to distributions with a peak at ω≈0, i.e., small turning angles, denoting a high persistence. When turning angles are mostly uniformly distributed, $g_{\omega }\approx 0$, and values close to $g_{\omega }\approx -1$ correspond to distributions peaked at large values of ω, close to ±π, as in U-turns. Mean sine $s_{\omega }$ describes the asymmetry of the distribution, with positive values indicating a bias for turning left, and negative values indicating a bias for turning right.

### Segment length distribution

The survival curves of the free path lengths in the different angular sectors fit an exponential distribution when segment lengths greater than 10 mm are considered, see Figs S1.3, S1.4, S1.9 and S1.10 in the S1 File. Small segment lengths are dominant in exponential distributions, but they are not accessible experimentally and the segmentation process further rarefies them. We therefore estimated mean free path λ for each angular sector from the linear part of the segment length survival curve (above length 10 mm) on a log-linear scale as the inverse of the weighted linear regression slope (absolute value) [26].

### Speed distribution

Since most distributions of speed values were skewed to small values (Fig S1.5, S1.6, S1.11 and S1.12 in S1 File) we chose the median speed $v_{med}$ instead of the mean to characterise the central tendency of the speed distributions.

Search behaviour may have different impacts on these quantities. One expects for example that when the animal is heading towards the release point, $g_{\omega }$ should be larger than when heading away. Also, when the heading is perpendicular to $\vec{u}$ we expect $s_{\omega }$ to be different from 0, biasing turning angles towards $\vec{u}$. These trajectory-wise parameters will be used for the estimation of directional effect sizes (see below).

Assembling all *l*, *v*, and ω values over all original trajectories, either in the $\Phi_{x}$ or $\Phi_{\vec{u}}$ approach, permits us to reconstruct their overall empirical distributions with respect to the orientation sector (see Figs S1.1-S1.12 in the S1 File). The overall values of $g_{\omega }$, $s_{\omega }$, λ, and $v_{med}$ are calculated from these assembled empirical distributions to plot their values as a function of each segment sector (in the same way as in [13] to simplify the comparison with their results). These empirical distributions are also used to simulate trajectories non-parametrically to assess the impact of the $\Phi_{x}$ or $\Phi_{\vec{u}}$ approaches on ant dispersal in a more classical drift-diffusion framework [9]. A simulated trajectory begins at the point (0,0) with the first segment heading drawn from a uniform angular distribution. The successive segment lengths and turning angles are then drawn from the empirical distributions corresponding to the orientation sector containing the current ant heading. In the $\Phi_{x}$ approach, the headings are computed with respect to the *x*-axis, while in the $\Phi_{\vec{u}}$ approach they are computed with respect to $\vec{u}$ that always points to the origin (0,0). In a first series of simulations, trajectories were simulated for 10s to assess the resulting spatial ant distribution. In a second series they were simulated until the ants cross a circle of diameter 200 mm around the origin in order to assess the time it takes them to get that far away. In both cases we simulated a total of 10,000 trajectories to reconstruct either the ants’ spatial distribution or the time distribution to cross the circle. We compared the times to cross this circle in the two approaches by computing the ratio of the mean time in the $\Phi_{x}$ approach divided by the mean time in the $\Phi_{\vec{u}}$ approach. The associated standard error was computed by a non-parametric bootstrap with 100,000 bootstrap samples [27]. To compare these simulated times with the original trajectories, we first computed for each original trajectory the time needed by ants to move a net distance of 200 mm from the release point. We then computed the ratio of their mean time divided either by the mean time in the $\Phi_{x}$ simulations or by the mean time in the $\Phi_{\vec{u}}$ simulations (computing the associated standard errors also by a bootstrap).

To test whether visual cues influence the ant’s search behaviour we run a series of additional experiments similar to those carried out in [13]. We placed individual ants of three queenless colonies of *L. niger* in a 50 × 50 cm circular arena isolated from the surroundings’ visual cues in the experimental room by black curtains. The experiments took place in a temperature (25°C) and humidity (50%) controlled room. We used the same protocol as in [13]: place a new object in the colony, select with a tooth pick (rather than a paint brush as in [13] to allow using an unmarked support for each ant) an ant that explores this object, transfer the ant passively on its tooth pick to the arena center and let it descend spontaneously, film its path for 3 minutes or until it reaches the arena wall. We tracked the movement of 60 individual ants (20 from each colony), with each ant tested successively under white light (LED SuperSlim 20W, luminosity 1800–2000 lm or 150 W, from ONSSI) and red light (Lee Filters 787 Marius Red placed in front of the LED lights) conditions (paired design). Colony and first light condition (white or red) were randomized. The light intensity in the arena center was measured with a luxmeter. Under red light the intensity was 17.6±0.6 lx, while under white light it was 725±24.5 lx. Colony and light conditions were randomized. After each experiment, the arena was cleaned with alcohol to remove any potential chemical cues [22]. To film the arena from above, we used a Sony ZV-E10 camera with a frame rate of 25 frames per second. The videos were then analysed with a custom-made in-house software, called TOSIA, in order to track the ants and obtain fixed-time trajectories of the individuals’ movements. The segmentation of the trajectories and their analysis were carried out as described above.

To assess whether there was any directional bias in the median speed $v_{med}$ of ants, and in the mean cosine $g_{\omega }$ and mean sine $s_{\omega }$ of the distribution of turning angles between the segments composing each trajectory according to the direction in which the ants were walking, we used linear mixed models [28]. We compared the values of these variables when ants were moving away or towards their starting point (combining the 3 angular sectors centered on the + Y or Away direction and the 3 angular sectors centered on the -Y or Start direction, for the $\Phi_{x}$ and $\Phi_{\vec{u}}$ approach, respectively) and when they were moving perpendicular to the left or right of this direction (combining the 3 angular sectors centered on the + X or right direction and the 3 angular sectors centered on the -X or left direction, for the $\Phi_{x}$ and $\Phi_{\vec{u}}$ approach, respectively). Trajectory identity was entered in the model as a random effect to account for individual variability and repeated measurements. We used the functions of the *R* package lmerTest [29] to obtain the p-values of the mixed models via Satterthwaite’s degrees of freedom method. The 95% confidence interval of model coefficients and effect size was calculated with the R package emmeans [30]. The quality of the model was assessed with the *R* package performance [31] and DHARMa [32].

To investigate whether the length of the mean free path λ varies with the ants’ walking direction, we used a survival analysis regression model. In the same way as for $v_{med}$, $g_{\omega }$ and $s_{\omega }$, we compared the length of the mean free path when ants were moving away or towards their starting point and when they were moving perpendicular to the left or right of this direction. To account for the dependency in the values of the lengths of the mean free path belonging to the same trajectory we used a mixed effects Cox proportional hazard model (coxme package [33]) with trajectory identity entered as a random factor.

To account for multiple inference due to the eight tests run for each configuration ($\Phi_{x}$ approach, $\Phi_{\vec{u}}$ approach, white light, red light), we applied a Bonferroni correction and considered a configuration-wide significance threshold value of 0.006. For the median speed $v_{med}$, mean cosine $g_{\omega }$ and the mean sine $s_{\omega }$, the effect size (ES: difference between the means of the two compared heading directions) and its 95% confidence interval are reported in the result section for each statistically significant comparison, whereas for the mean free path λ the odds ratio (OR) and its confidence interval are indicated. When comparing two opposite directions A and B, an odds ratio higher than unity, e.g., 1.3, means that the probability for the length of the mean free paths to be longer in the A direction is 30% higher than that in the B direction.

The trajectory analysis, statistical comparisons and simulations, were all performed with the R software version 4.3 [34] in the RStudio IDE [35]. The corresponding scripts and the trajectories can be found in the open data repository Zenodo [36].

## Results

**Fig 2:**
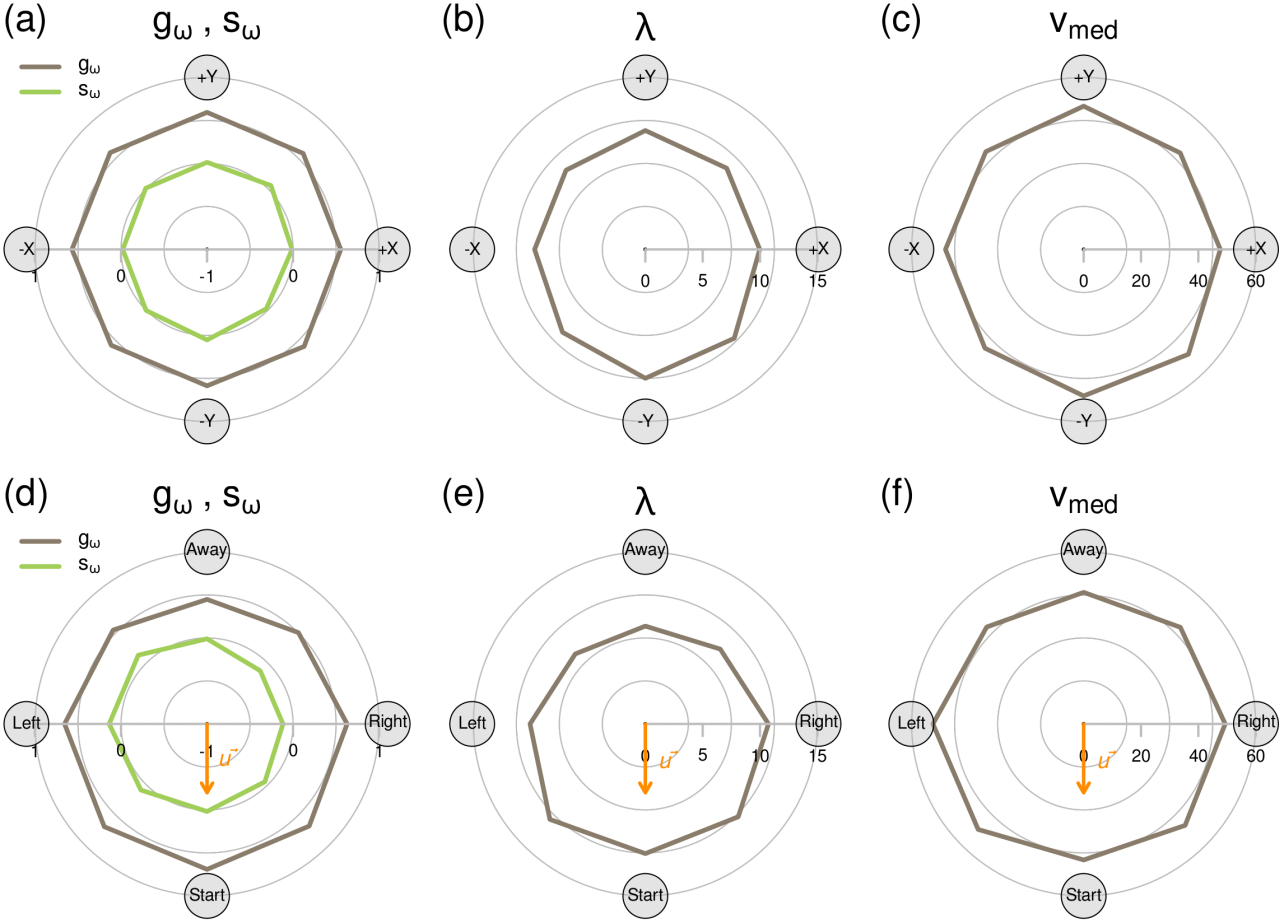
Estimated values (corners of the octagons) of the four Boltzmann random walker parameters $g_{\omega }$, $s_{\omega }$, λ, and $v_{med}$, as a function of the heading angle (grouped in eight sectors) calculated with respect to the camera’s *x*-axis in the $\Phi_{x}$ approach (a,b,c), and as a function of the heading angle of the path segments calculated with respect to $\vec{u}$ pointing to the starting point of the trajectory, $\Phi_{\vec{u}}$ approach, (d,e,f). All trajectories were cumulated, and the distance of a point (corner of an octagone) to the centre of the circles represents the overall estimated parameter value in each 45 ° sector (see scale along the *x*-axis). (a,d) Mean cosine $g_{\omega }$ (thick gray octagon), and mean sine $s_{\omega }$ (green octagon), (b,e) mean segment length λ (unit mm), (c,f) median speed $v_{med}$ (unit mm/s). Away refers to headings that make the ants move away from the starting point, Start to headings that make them come back towards this point (in the $\vec{u}$ direction). See Figs S1.1-S1.6 in S1 File for the detailed associated distributions.

Fig 2 summarizes the estimated Boltzmann walker parameters reconstructed as a function of the ant heading angle, either with respect to the camera’s *x*-axis ($\Phi_{x}$ approach) or with respect to the vector $\vec{u}$ pointing towards the starting point of the trajectories ($\Phi_{\vec{u}}$ approach). Although the same underlying values of *l*, $v_{med}$, $g_{\omega }$ and $s_{\omega }$ are used in both approaches, they are redistributed differently across angular sectors because the heading angle ϕ is computed either with respect to the fixed camera’s *x*-axis or with respect to the position-dependent vector $\vec{u}$ (Fig 1). As a result, orientation-dependent effects that are averaged in the $\Phi_{x}$ representation become apparent in the $\Phi_{\vec{u}}$ framework.

When distributions are built in the $\Phi_{x}$ approach (Fig 2a-2c), all corresponding parameters look isotropic, there is no effect of orientation on their values (compare to Fig 5 in [13]). Regarding the mean cosine $g_{\omega }$ and mean sine $s_{\omega }$, the $\Phi_{x}$ approach (Fig 2a, Fig S1.1 and Tables S1.1 and S1.2 in S1 File) yielded no significant bias either in the + Y/-Y direction or in the + X/-X direction. However, in the $\Phi_{\vec{u}}$ approach (Fig 2d, Fig S1.2 in S1 File) we detected a significant bias in the Start direction for the mean cosine $g_{\omega }$ (Table S1.1 in S1 File, *F*(1, 336.5)= 78.62, *p* < 0.001). Ants coming back to the starting point have a significantly narrower turning angle distribution (ES($g_{\omega }$)= −0.14 [−0.17 −0.11]). In addition, when ants move perpendicular to $\vec{u}$, they have a tendency to turn back towards their starting point (Table S1.2 in S1 File: differences in mean sine $s_{\omega }$: *F*(1, 193.1)= 67.40, *p* < 0.001). The associated distribution of $s_{\omega }$ is asymmetric: left 0.11 [0.07 0.14], right −0.09 [−0.12 −0.05].

For median speed $v_{med}$, both the $\Phi_{x}$ (Fig 2c, Fig S1.5 in S1 File) and the $\Phi_{\vec{u}}$ (Fig 2f, Fig S1.6 in S1 File) approach showed no significant bias according to walking direction (Table S1.1 and S1.2 in S1 File).

The mixed Cox model revealed no significant bias in the distribution of mean free path lengths λ in the $\Phi_{x}$ approach (Table S1.3 in S1 File, Fig 2b, Fig S1.3 in S1 File). On the other hand, we found a significant effect of walking direction on the distribution of mean free path lengths in the $\Phi_{\vec{u}}$ approach (Table S1.3 in S1 File, Fig 2e, Fig S1.4 in S1 File: z=−6.91,p<0.001): the mean free paths were shorter when ants move away from their starting point than when they move towards it (start direction, OR(λ)= 0.46 [0.37 0.58]), indicating a strong anisotropy in movement behavior.

**Fig 3:**
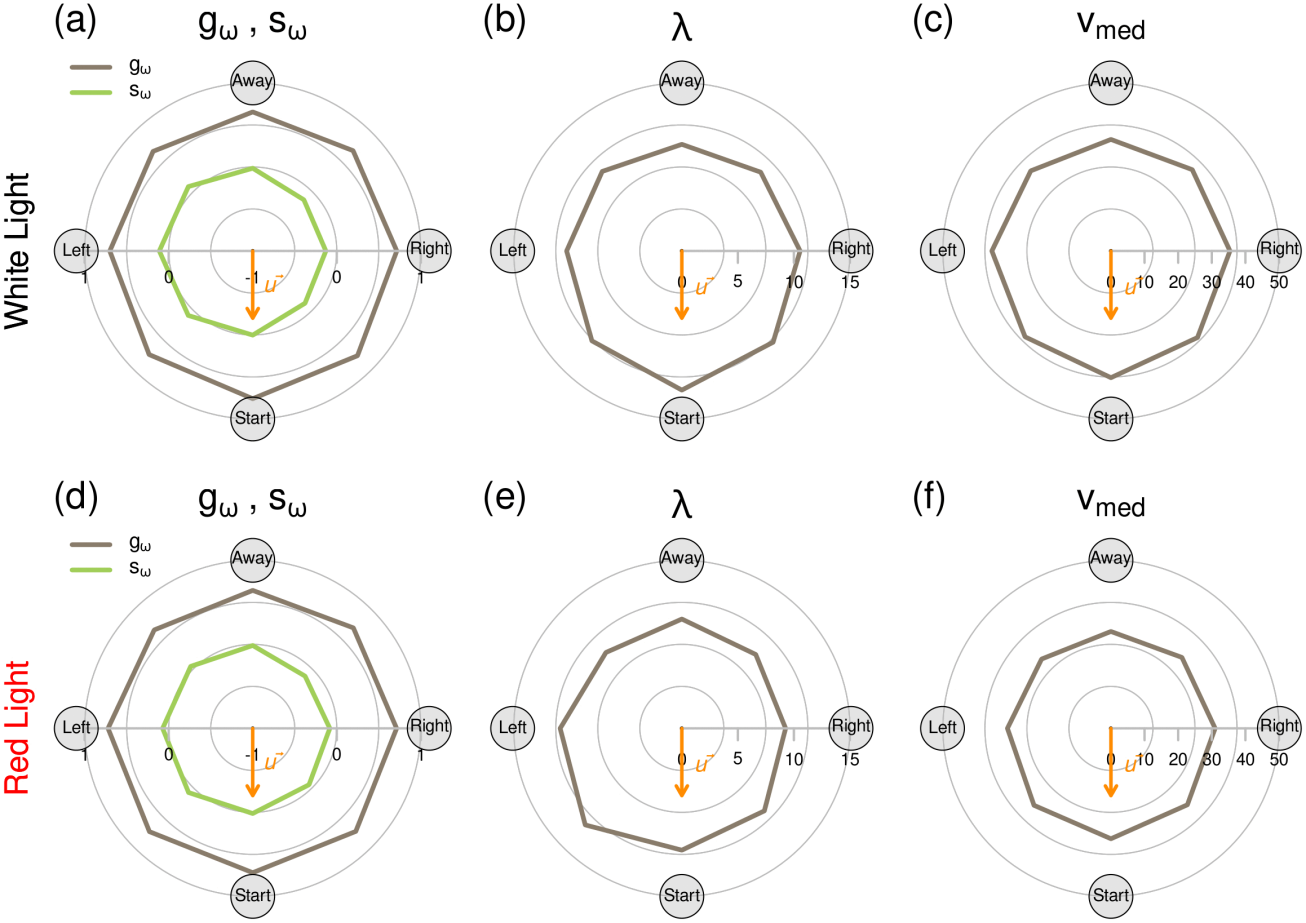
Estimated values of the four Boltzmann random walker parameters $g_{\omega }$, $s_{\omega }$, λ, and *v*, calculated in the $\Phi_{\vec{u}}$ approach (i.e., heading angles of the path segments, calculated with respect to $\vec{u}$ pointing to the starting point of the trajectory), for trajectories under white (a,b,c) and red light (d,e,f). The distance of each point (corner of the octagon, at the center of each 45° angular sector) to the center of the circles represents the mean parameter value over *n* = 60 trajectories (see scale along the *x*-axis). (a,d) Mean cosine $g_{\omega }$ (thick grey octagon), and mean sine $s_{\omega }$ (green octagon), (b,e) mean segment length λ (unit mm), (c,f) median speed $v_{med}$ (unit mm/s).

Fig 3 shows the results of our own experiments under white light and under red light using the $\Phi_{\vec{u}}$ approach.

Under white light, as in the $\Phi_{\vec{u}}$ approach in Fig 2, ants that move perpendicular to $\vec{u}$ have a tendency to turn back towards their starting point ($s_{\omega }$: Fig 3a, Fig S1.7 and Table S1.2 in S1 File, *F*(1, 158.2)= 39.81, *p* < 0.001, left: 0.07 [0.02 0.11], right: −0.09 [−0.14 −0.05]). There was also a significant bias in the median walking speed $v_{med}$ in the Away/Start direction (Fig 3c, Fig S1.11 and Table S1.1 in S1 File: *F*(1, 271.6)= 34.172, *p* < 0.001): ants walk more slowly when they move away from their starting point (ES = −2.27 [−3.03 −1.50] mm/s). Finally, the length of the mean free path λ, although above the configuration-wide significance threshold, was slightly shorter when ants walk away from their starting point (Fig 3b, Fig S1.9 and Table S1.3 in S1 File: z=−2.03, *p* = 0.042, OR(λ)= 0.78 [0.62 0.99]).

Under red light, no significant biases in either the Away/Start or Left/Right direction were detected for the directional persistence $g_{\omega }$ and mean sine $s_{\omega }$ (Fig 3d, Fig S1.8 and Tables S1.1-2 in S1 File), and for the mean free path λ (Fig 3e, Fig S1.10 and Table S1.3 in S1 File). The median speed $v_{med}$ was significantly influenced by orientation (Fig 3f, Fig S1.12 and Table S1.1 in S1 File). Ants walk faster towards their starting point than away from it: *F*(1, 279.7)= 16.42, *p* < 0.001, ES = −1.70 [−2.53 −0.88] mm/s.

See Fig S1.15 in S1 File for a sample of trajectories observed in the three configurations studied.

**Fig 4:**
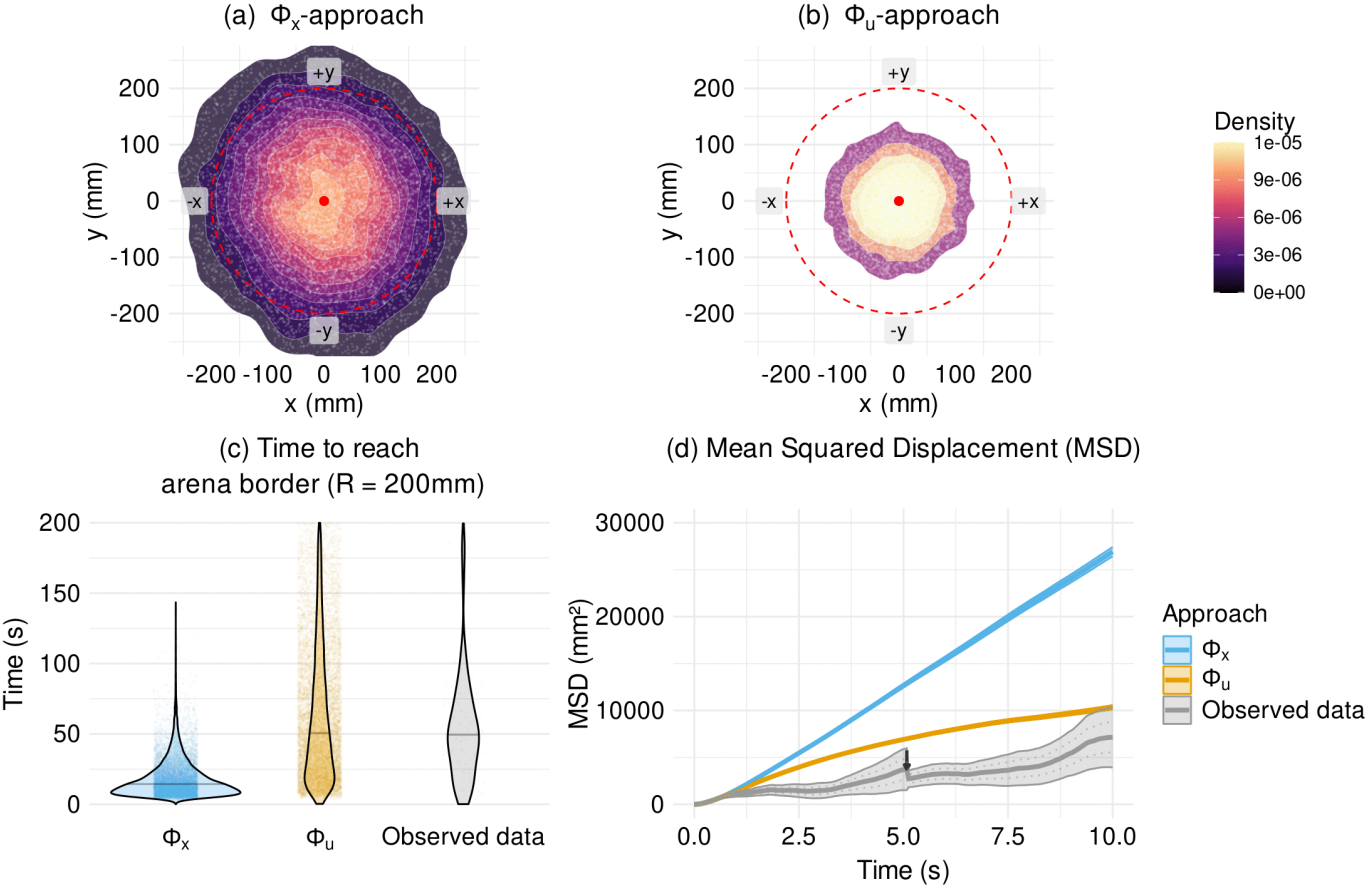
Impact of the $\Phi_{x}$ (a) and $\Phi_{\vec{u}}$ (b) approach on ant movement assessed by non-parametric simulations based on the empirical distributions of *l*, *v* and ω that were reconstructed either in the $\Phi_{x}$ or the $\Phi_{\vec{u}}$ approach from the Khuong *et al* data (Fig 2, see M&M for details). (a,b) Density plot (with contour lines) of ants after a 10s path simulated from the same point (red central dot). White refers to high density, black to low density. The red dotted circle shows the 200 mm net displacement limit to compute the times in Fig (c). In the $\Phi_{x}$ approach (a) ant orientation is assessed with respect to the camera *x*-axis while in the $\Phi_{\vec{u}}$ approach (b) it is assessed with respect to the vector $\vec{u}$ (considered to point always to the red central dot). (c) shows the violin plots of times to perform a net displacement of 200 mm for these simulations (red dashed circles in (a,b)) and the experimental data. (d) shows mean net squared displacement (MSD) for the two simulations (straight blue line for the $\Phi_{x}$ approach, sub-diffusive curved orange line in the $\Phi_{\vec{u}}$ approach) and the experimental data (mean and CI_95%_). The black arrow indicates the time where the first trajectory hit the arena border and no longer contributed to MSD computation.

At this stage, the question arises whether the small observed effects of heading upon the mean cosine $g_{\omega }$, mean sine $s_{\omega }$, median speed $v_{med}$, and mean free path λ in the $\Phi_{\vec{u}}$ approach would be sufficient to yield a change on ant dispersal. For testing this, and while waiting for a full formalisation of the Generalised Boltzmann Walker model embedding the $\Phi_{\vec{u}}$ approach, we explore this question numerically with non-parametric path simulations based on the reconstructed empirical distributions of the Boltzmann walker variables (Figs S1.1-S1.6 in S1 File). In the $\Phi_{x}$ approach, ants disperse equally in all directions (Fig 4a), and the distribution of final positions tends to some Gaussian shape, as expected under pure diffusion, with its variance becoming proportional to time (linear MSD in Fig 4d). By contrast, in the $\Phi_{\vec{u}}$ approach, the simulated ants’ positions show a strong tendency to remain close to their initial position (Fig 4b), as expected under drift-diffusion, with MSD becoming bounded (or sub-diffusive, Fig 4d). Hence, the effect sizes on mean free path, $g_{\omega }$, $s_{\omega }$ and median speed at the segments’ scale are by far enough to yield a major change on ant dispersal. This bias also influences the time to move at least 200 mm away from the starting point (Fig 4c), which is 4.06 (± 0.05 se) times longer in the $\Phi_{\vec{u}}$ approach compared to the $\Phi_{x}$ approach. The times ants took to move this net distance in the experiments are very close to the $\Phi_{\vec{u}}$ simulated times (the ratio of experimental mean times divided by simulated mean times is 0.83 ± 0.10 se), but much longer than the $\Phi_{x}$ simulated times (the ratio of experimental mean times divided by simulated mean times is 3.37 ± 0.41 se). Note also that experimental MSD is much closer to that predicted by the $\Phi_{\vec{u}}$ approach compared to that predicted by the $\Phi_{x}$ approach (orange and blue lines in Fig 4d respectively).

## Discussion

In this paper we reanalysed the data of the ants from Khuong *et al.* (2013) [13] moving on a novel area, consisting of a flat homogeneous surface, to which they have been passively displaced. After analysing the ant trajectories the authors were led to the conclusion that the random walk parameters describing the ants’ trajectories were homogeneous in space. We questioned this conclusion and hypothesized that ants are able to assess their location in space and that their trajectories are biased towards their starting point when exploring a new area. We thus re-calculate the random walk parameters of their trajectories as a function of their orientation with respect to the starting point of their trajectory. We found that ants have a strong tendency to remain close to the starting point of their exploration when exploring a new area, i.e., that they perform a search behavior alike that described in other ant species when searching for their nest or a predictable food source [37]. The literature already suggests that *L. niger* ants can perform search behavior around the previous location of a food source in presence of visual landmarks [38], but our analysis provides evidences that they can also do so in the absence of visual cues, under red light, when exploring a novel area.

To perform this search behaviour we found that the ants’ heading with respect to their starting point influences all parameters involved in a correlated random walk (or Boltzmann walk): their speed *v* (faster when heading back), free path λ (longer when heading back), turning angle parameters mean cosine $g_{\omega }$ (smaller turning angles when heading back) and mean sine $s_{\omega }$ (tendency to turn towards back direction). These findings apply both to the data from Khuong et al [13] and to our own data from experiments run in similar conditions under white light. Under red light, on the other hand, the distribution of the turning angles did not change and only the ant speed and the length of their mean free path was influenced by the ants’ heading relative to their starting point.

*L. niger* ants are known to use visual cues to navigate in their environment [18,19,21]. In our experiment, the area on which ants were moving during the tests was isolated visually from the experimental room with black sheets. However, these sheets did not diffuse light in an homogeneous manner; ants may thus be able to locate the approximate location of the starting point of their trajectory through a matching process between a view stored at this point and their current view of the arena [39]. This may explain how they are able to perform a biased random walk towards their starting point under white light. However, we detected the same type of search behaviour even under red-light conditions, indicating that ants do not rely solely on vision for their orientation (provided that the red light we used cannot be perceived by *L. niger* [40]). In the absence of visual cues one could hypothesize that ants could orient on chemical marks forming a gradient centered on the starting point of their trajectories. However, *L. niger* lays trail pheromone only after the discovery of a food source [41] and the amount of home range marking passively deposited through footprints on a novel area is probably too small to create a gradient [22]. Another hypothesis could be that ants rely on path integration to keep track of the location of the starting point of their trajectory. Although this navigation mechanism has so far been demonstrated in ants only in presence of an external compass [11], one cannot rule out that, as recently shown in the fruit fly *Drosophila melanogaster* [12] moving in complete darkness, they could use path integration based on idiothetic cues when visual cues are absent. Further research would be needed to determine whether this is the case.

It is not an easy task to obtain general predictions from random walk model formulations (see review in [9]). One solution is the passage to macroscopic equations that are more accessible to mathematical analysis. The Boltzmann walker modeling framework can directly be translated into a transport equation which permits to derive associated partial differential equation systems [42,43]. The case of Khuong *el al*’s analysis on a flat horizontal surface corresponds to a correlated random walk (CRW, the model parameters found are isotropic, they do not depend on ant orientation). The associated passage to macroscopic diffusion type models can be found in [42], or in the appendix in [44] which also discusses whether the approximations that are made in the process are pertinent in the biological situation at hand. The case of the CRW leads to the classical telegraph equation if we only make the so-called P1-approximation (which applies when the spatial scale of interest requires many turning events [45]), or to the standard Fickian diffusion equation if we also make the diffusion approximation (flux density dynamics can be considered stationary compared to animal density dynamics, which applies when mean free paths are much smaller than the spatial scales of any environmental heterogeneity that influences mean free paths). In both cases the macroscopic model predicts that average ant density evolves as a Gaussian distribution around the starting point, with a standard deviation that is proportional to the square root of time. Ants therefore can move arbitrarily far away from the starting point as time goes on (mean net squared displacement increases linearly with time [17]). This is predicted by our non-parametric simulations in the $\Phi_{x}$ approach (Fig 4d, blue line), but this pattern is clearly not seen in our data (see experimental MSD in Fig 4d): in Fig S1.13 in S1 File we pushed the non-parametric simulations from Fig 4 to longer times (up to 200 s). We see that in the $\Phi_{\vec{u}}$ simulations a stationary area-restricted search is achieved after 50 s, with a simulated MSD that is very close to the experimental MSD. On the contrary, the MSD of the $\Phi_{x}$ simulations quickly diverges from the experimental MSD. With our detected dependence of model parameters (form of the turning angle distribution, mean speed, mean segment length) on the animal’s orientation with respect to the direction $\vec{u}$ from its current position to its initial location, the Boltzmann walker actually becomes a biased correlated random walk BCRW (sensu [9], especially their section 3). The predicted MSD from our $\Phi_{\vec{u}}$ simulations (Fig S1.13 in S1 File) can be found in standard advection-diffusion models [46]. However, the passage from this BCRW to macroscopic models has yet to be made, in particular to assess whether the approximations made in the process apply to ant searching behaviour [45].

To further illustrate the impact of the detected BCRW movement on ant dispersal we also explore the case where animals begin their search far from a target location. This may happen when ants are passively displaced by wind [47] or when they are accidentally released while being passively transported by a conspecific [48]. To simulate trajectories in that case we use the empirical distributions reconstructed in the $\Phi_{\vec{u}}$ approach, but during the trajectory progression we assess animal orientation with respect to a fixed γ direction ($\Phi_{\vec{\gamma }}$ simulations, Fig. S1.14d in S1 File), as if the target location were located at an infinite distance in this γ direction, see Fig S1.14 in S1 File and simulated example trajectories in Figs. S1.16-S1.18 in S1 File. In that case ants move in a super-diffusive way in the γ direction (Fig S1.14c, in S1 File, the green curve is well above the diffusive blue curve) and they perform a 200 mm net displacement 1.61 (± 0.01 se) times faster than in the $\Phi_{x}$ approach (Fig S1.14b in S1 File), mostly in the γ direction (Fig S1.14a in S1 File).

These non-parametric simulations clearly illustrate that the detected subtle changes in the random walk parameter values can have a profound impact on ecologically relevant statistics such as average dispersal (MSD) or return times towards a target point. Further theoretical investigations, either at the individual based model level or at the macroscopic level (and the mathematical passage to get there) will prove useful to better assess such effects for a given experimental or field situation.

In conclusion, our analyses show that *L niger* ants display search behaviour when exploring an unknown environment to which they have been passively displaced. They retain this search behaviour even under red-light conditions, showing that vision is not the only sense used to perform search behaviour. At the macroscopic level this indicates that ant dispersal does not proceed as predicted by simple diffusion models, but that advective type dispersal emerges, permitting ants to stay close to their initial position, even on long time scales, or to return quickly to their target location if they find themselves far away from it. The impact of this search behaviour on colony level functions will require further investigations.

## Supporting information

S1 FileMain analyses supplement.Detailed reconstructed distributions of the Boltzmann walker variables *l*, *v* and ω summarised in Figs 2 and 3 (Figs. S1.1-S1.12); extended non-parametric simulation work complementary to Fig 4 (Figs. S1.13 + S1.14); examples of experimental and simulated trajectories (Figs. S1.15-S1.18); tables of effect sizes estimated in the different statistical analyses (Tables S1.1-S1.3).(PDF)
