# Supplementary material for: Discovering search behaviour in black garden ant trajectories
Source: PLoS One. 2026 Jul 29;21(7):e0327957. doi: 10.1371/journal.pone.0327957 (PMC13419209; doi:10.1371/journal.pone.0327957)
Supplement: S1 File — Detailed reconstructed distributions of the Boltzmann walker variables l, v and ω summarised in Figs 2 and 3 (Figs. S1.1-S1.12); extended non-parametric simulation work complementary to Fig 4 (Figs. S1.13 + S1.14); examples of experimental and simulated trajectories (Figs. S1.15-S1.18); tables of effect sizes estimated in the different statistical analyses (Tables S1.1-S1.3). (PDF) [file pone.0327957.s001.pdf]

S1 File. Main analyses supplement.

Perrine Bonavita[1,3]◑*, Marius Albino[1]◑, Jacques Gautrais[1,2], Vincent Fourcassié[1,2], Maud Combe[1,2], Loic Lacour[1,2], Simon Eibner[3‡], Christian Jost[1,2,‡*]

1 Centre de Recherches sur la Cognition Animale, Université Paul Sabatier, Université Fédérale de Toulouse Midi-Pyrénées, Toulouse, cedex 9, France,
2 CNRS—Centre de Recherches sur la Cognition Animale, UMR 5169, 118 route de Narbonne, Toulouse, cedex 9, France
3 Department of Energy and Process Engineering, Centre RAPSODEE, IMT Mines Albi, Albi, Occitanie, France

◑These authors contributed equally to this work. ‡These authors also contributed equally to this work.
* `christian.jost@utoulouse.fr` or `bonavitaperrine@gmail.com`

List of Figures

S1.1 Khuong *et al* data, Φ_x approach, ω_i distributions, g_ω, s_ω . . . 2
S1.2 Khuong *et al* data, $\Phi_{\vec{u}}$ approach, ω_i distributions, g_ω, s_ω . . . 3
S1.3 Khuong *et al* data, Φ_x approach, l_i distributions, λ . . . 4
S1.4 Khuong *et al* data, $\Phi_{\vec{u}}$ approach, l_i distributions, λ . . . 5
S1.5 Khuong *et al* data, Φ_x approach, v_i distributions, median speed v_{med} . . . 6
S1.6 Khuong *et al* data, $\Phi_{\vec{u}}$ approach, v_i distributions, median speed v_{med} . . . 7
S1.7 White light data, $\Phi_{\vec{u}}$ approach, ω_i distributions, g_ω, s_ω . . . 8
S1.8 Red light data, $\Phi_{\vec{u}}$ approach, ω_i distributions, g_ω, s_ω . . . 9
S1.9 White light data, $\Phi_{\vec{u}}$ approach, l_i distributions, λ . . . 10
S1.10 Red light data, $\Phi_{\vec{u}}$ approach, l_i distributions, λ . . . 11
S1.11 White light data, $\Phi_{\vec{u}}$ approach, v_i distributions, median speed v_{med} . . . 12
S1.12 Red light data, $\Phi_{\vec{u}}$ approach, v_i distributions, median speed v_{med} . . . 13
S1.13 Ant dispersal on longer time scales: comparison between Φ_x and $\Phi_{\vec{u}}$ approach . . . 14
S1.14 Ant dispersal far from the starting point: $\Phi_{\vec{\gamma}}$ approach . . . 15
S1.15 Examples of experimental trajectories . . . 16
S1.16 Examples of simulated trajectories Khuong data . . . 17
S1.17 Examples of simulated trajectories Red Light data . . . 18
S1.18 Examples of simulated trajectories White Light data . . . 19

List of Tables

S1.1 Away *vs.* Start: mixed model comparison and effect size statistics for v_{med}, g_ω, s_ω . . . 20
S1.2 Left *vs.* Right: mixed model comparison and effect size statistics for v_{med}, g_ω, s_ω . . . 21
S1.3 Mixed Cox model analyses for Mean free path λ . . . 22

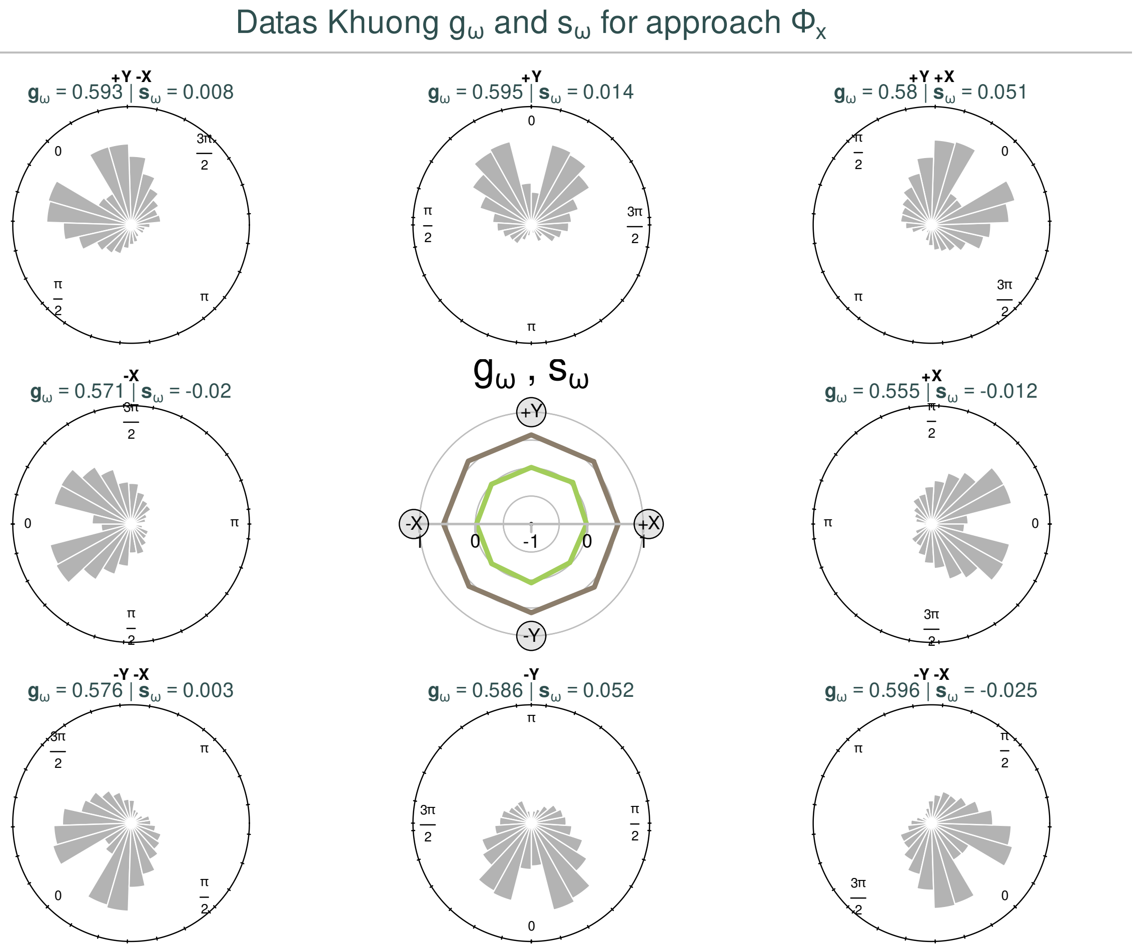

Fig S1.1. Khuong *et al* data: Empirical turning angle distributions as a function of ant orientation with respect to the camera's x-axis (Φ_x-approach). The central Figure reproduces the plot of the mean cosine and mean sine from the main text Fig. 2a, showing these parameters for each of the 8 angular orientation sectors (see main text Material & Methods). The eight other figures show the empirical distributions (for all the trajectories combined) as polar plots (the 0 direction indicates the sector's central direction). The rarity of small turning angles results from the segmentation algorithm. In this approach ant heading has no influence on the turning angle distributions, all look symmetrical (mean sine 0) and have the same forward tendency (same mean cosine).

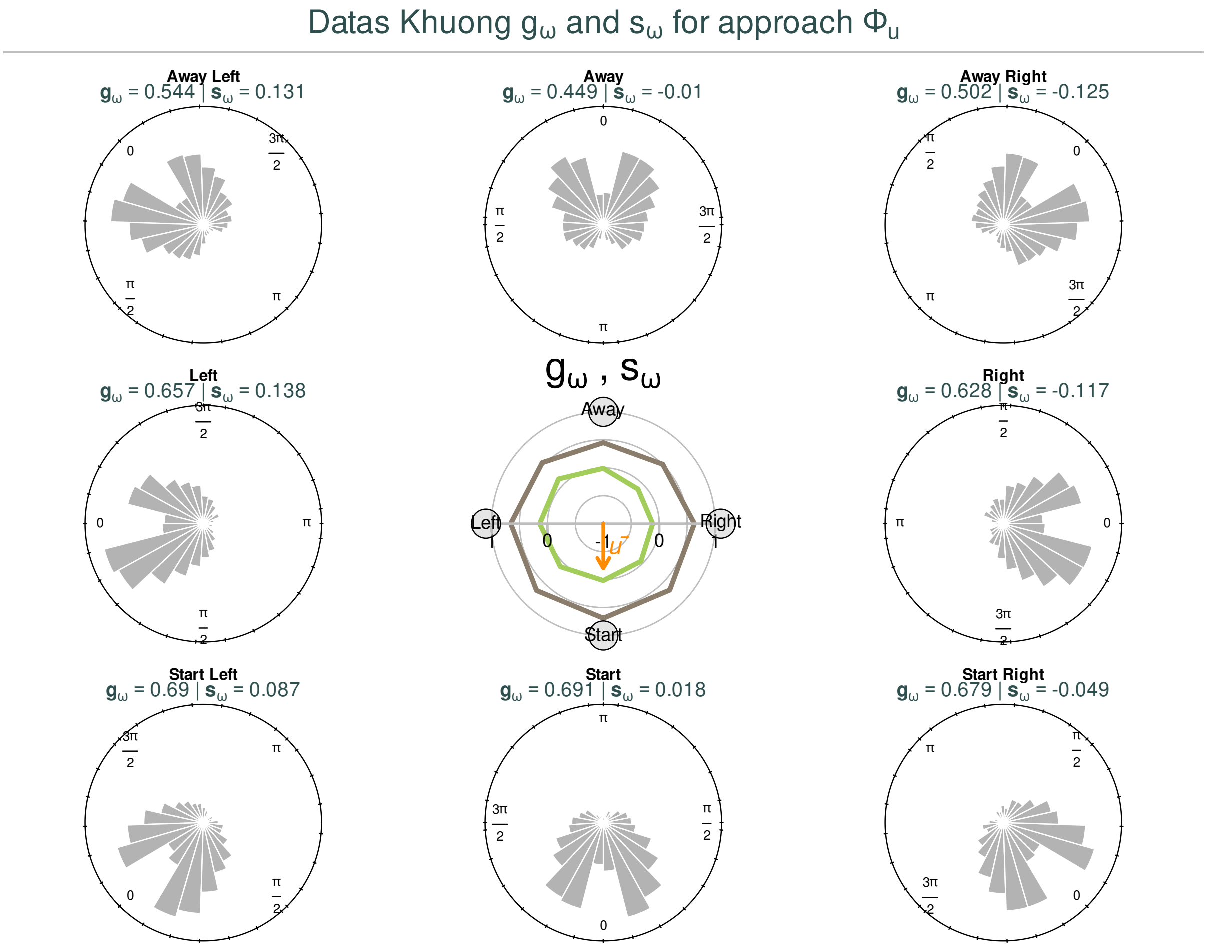

Fig S1.2. Khuong *et al* data: Empirical turning angle distributions as a function of ant orientation with respect to the $\vec{u}$ direction pointing towards the starting point of the trajectory ($\Phi_{\vec{u}}$-approach), organized as in Fig. S1.1. The central Figure reproduces the plot of the mean cosine and mean sine from the main text Fig. 2d. Ants moving away from the starting point have a less peaked distribution than ants moving back to the starting point. Ants with negative orientations with respect to $\vec{u}$ (left part of this figure) have a tendency to turn to the left (towards the starting point), and ants with positive orientations with respect to $\vec{u}$ tend to turn to the right (also towards the starting point).

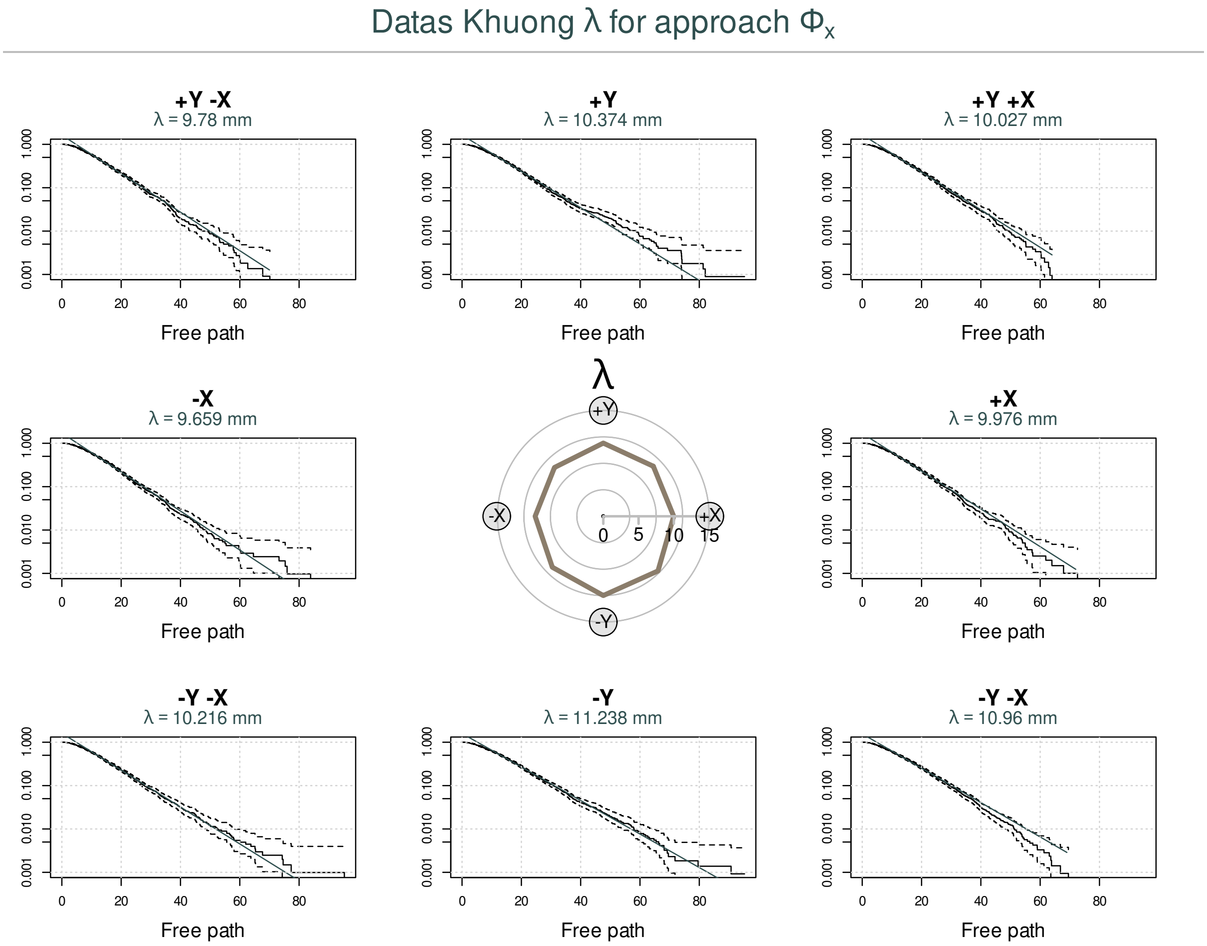

Fig S1.3. Khuong *et al* data: Survival curves (solid curve, the dotted lines indicate a 95% confidence band, log-linear scale) of free paths l as a function of ant orientation sector with respect to the camera's x-axis (Φ_x-approach). The straight lines show the weighted linear regressions (fitted to the survival curve values $>$ 10mm) to estimate the slope (its absolute value is the inverse of the mean free path λ). The central Figure is the same as the main text Fig. 2b, showing these mean free paths for each of the 8 angular orientation sectors (see main text Material & Methods). All λ-values are similar (do not depend on orientation, isotopic).

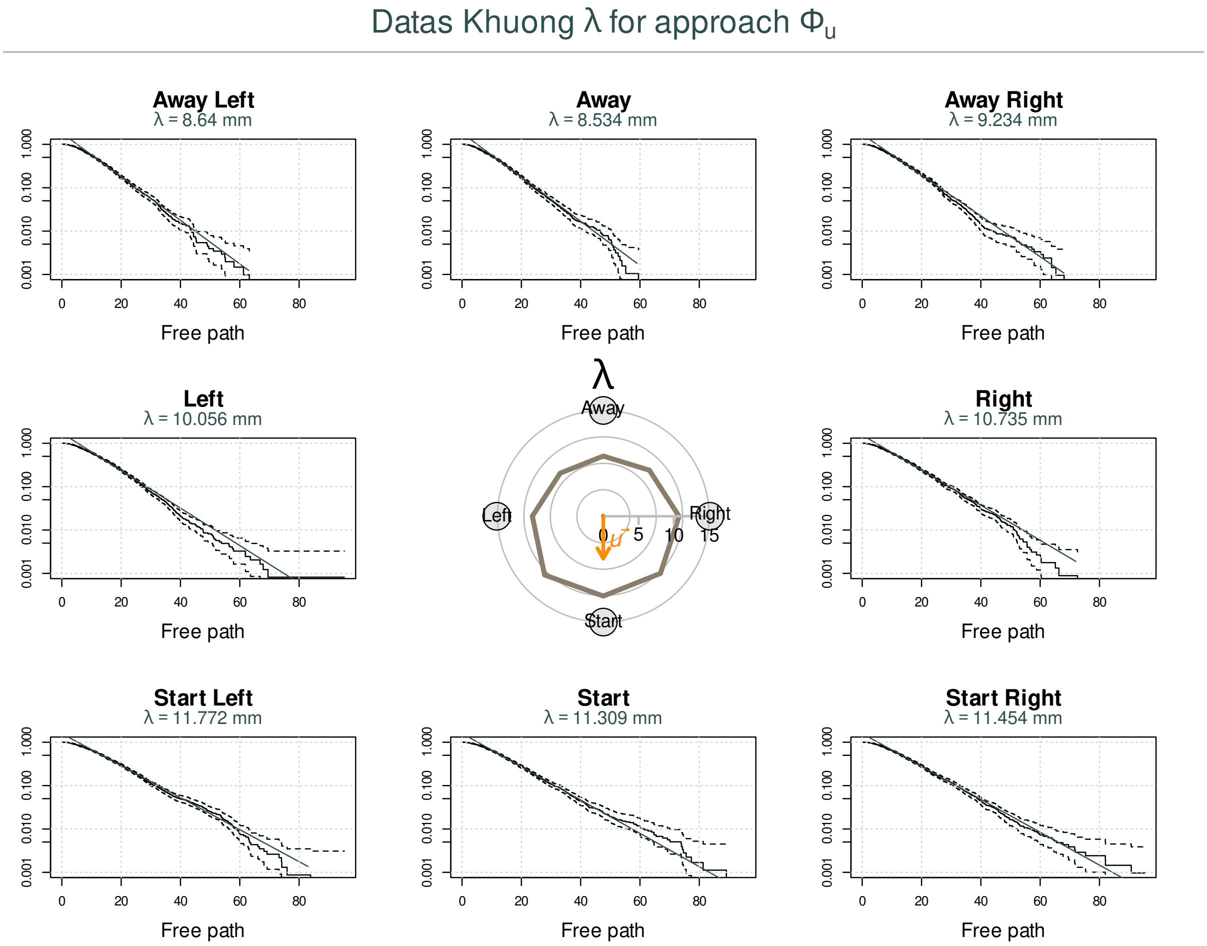

Fig S1.4. Khuong *et al* data: Survival curves of free paths l as a function of ant orientation with respect to the $\vec{u}$ direction pointing back to the starting point ($\Phi_{\vec{u}}$-approach), organized as in Fig. S1.3. The straight lines show the weighted linear regressions (fitted to the survival curve values > 10mm) to estimate the slope (its absolute value is the inverse of the mean free path λ). The central Figure is the same as the main text Fig. 2e, showing mean free path λ as a function of ant orientation. Mean free paths moving back to the starting point are longer than those moving away, but there is left-right symmetry.

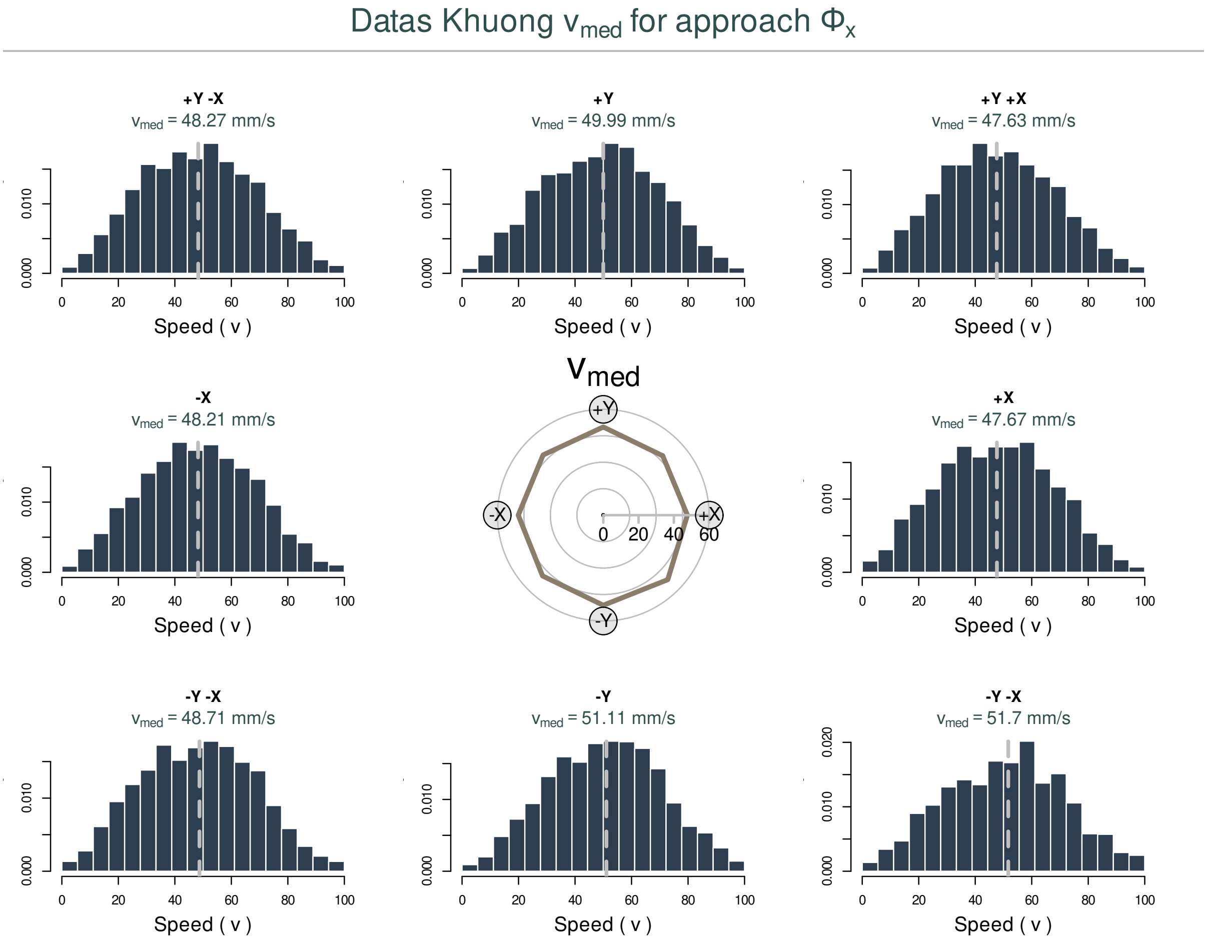

Fig S1.5. Khuong *et al* data: Empirical ant speed v (computed for each free path l) distributions as a function of ant orientation sector with respect to the camera's x-axis (Φ_x-approach) (the central Figure is the same as main text Fig. 2c, showing median speed v_{med} as a function of ant orientation sector). Median speeds v_{med} are similar in all orientation directions, speeds v ranging from 0 to 100 mm/s.

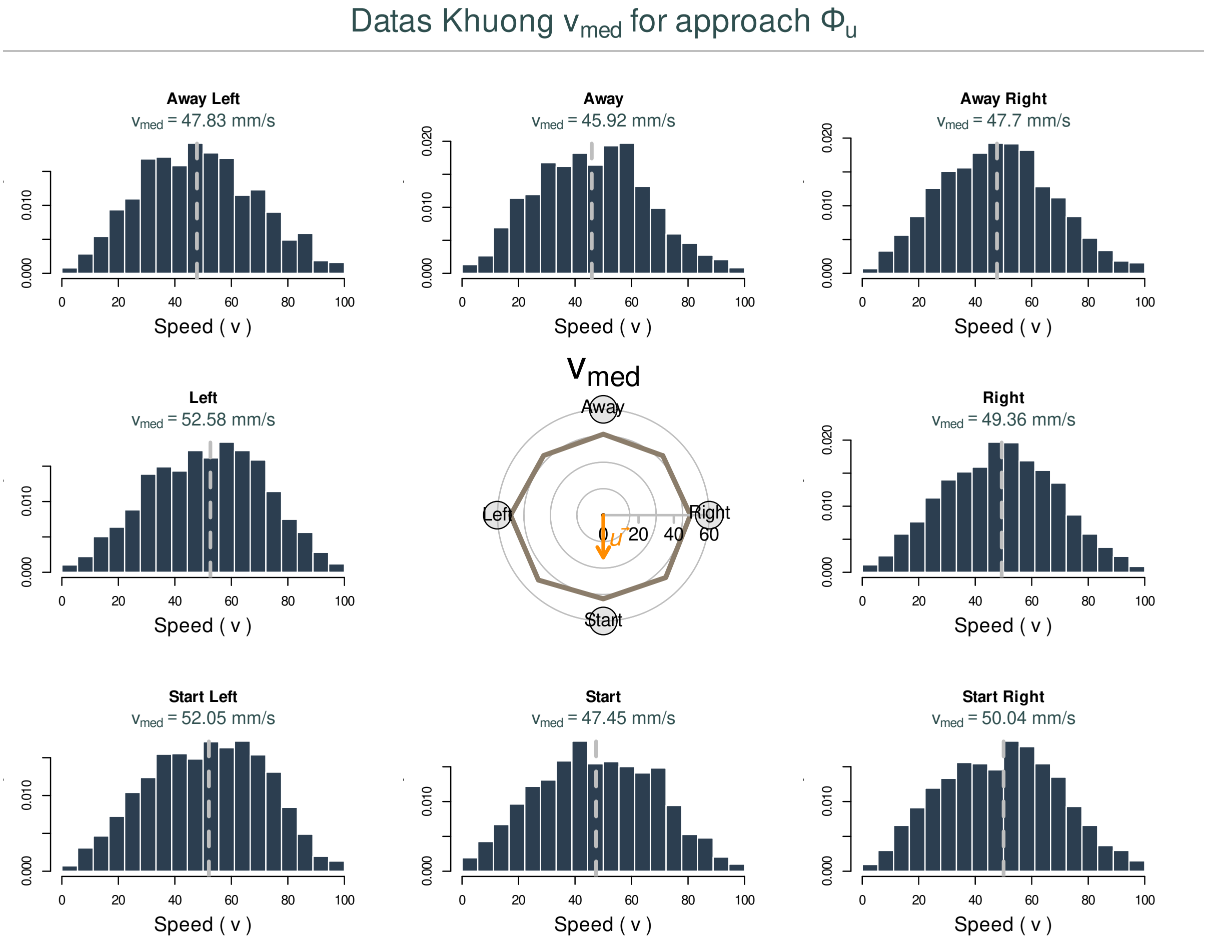

Fig S1.6. Khuong *et al* data: Empirical ant speed v (computed for each free path l) distributions as a function of ant orientation sector with respect to the $\vec{u}$ direction pointing back towards the starting point ($\Phi_{\vec{u}}$-approach), organized as in Fig. S1.5 (the central Figure is the same as the main text Fig. 2f, showing median speed v_{med} as a function of ant orientation).

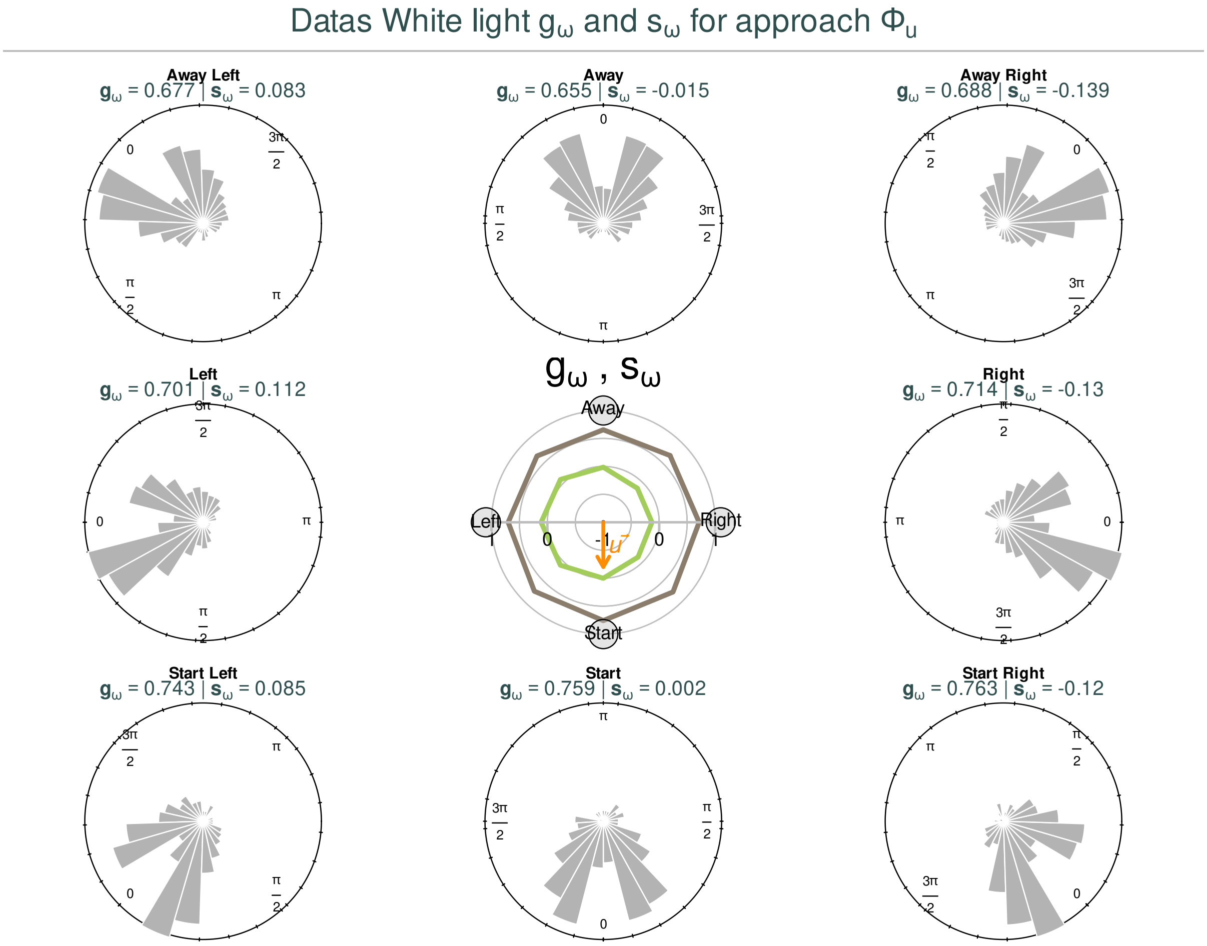

Fig S1.7. White light data: Empirical turning angle distributions as a function of ant orientation with respect to the $\vec{u}$ direction pointing towards the starting point of the trajectory ($\Phi_{\vec{u}}$-approach), organized as in Fig. S1.1, the central Figure is the same as main text Fig. 3a. For mean sine s_ω we detect the same tendenciy as in Khuong *et al*'s data in Fig. S1.2, a strong bias to turn towards the starting point.

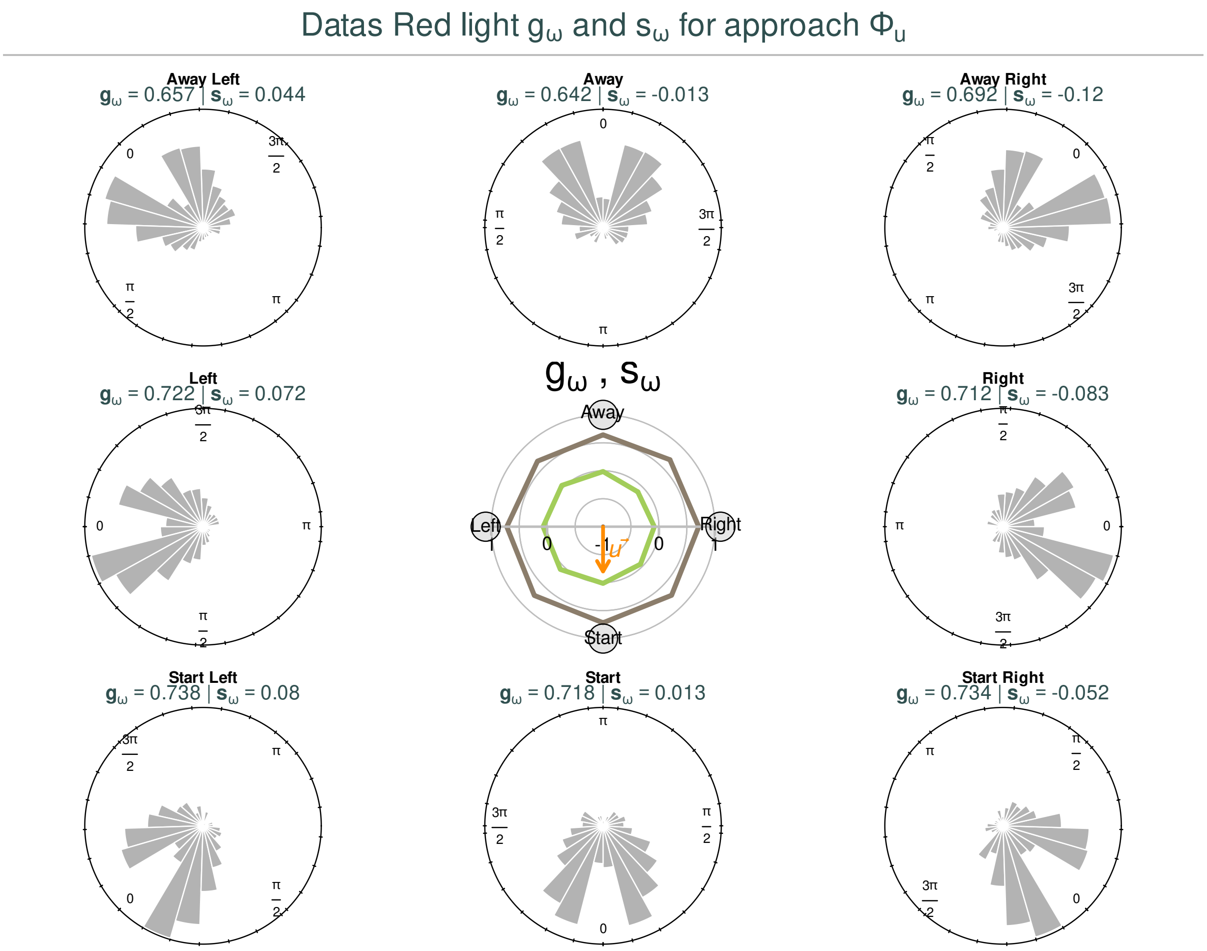

Fig S1.8. Red light data: Empirical turning angle distributions as a function of ant orientation with respect to the $\vec{u}$ direction pointing towards the starting point of the trajectory ($\Phi_{\vec{u}}$-approach), organized as in Fig. S1.2, the central Figure is the same as Fig. 3d. No directional biases were detected.

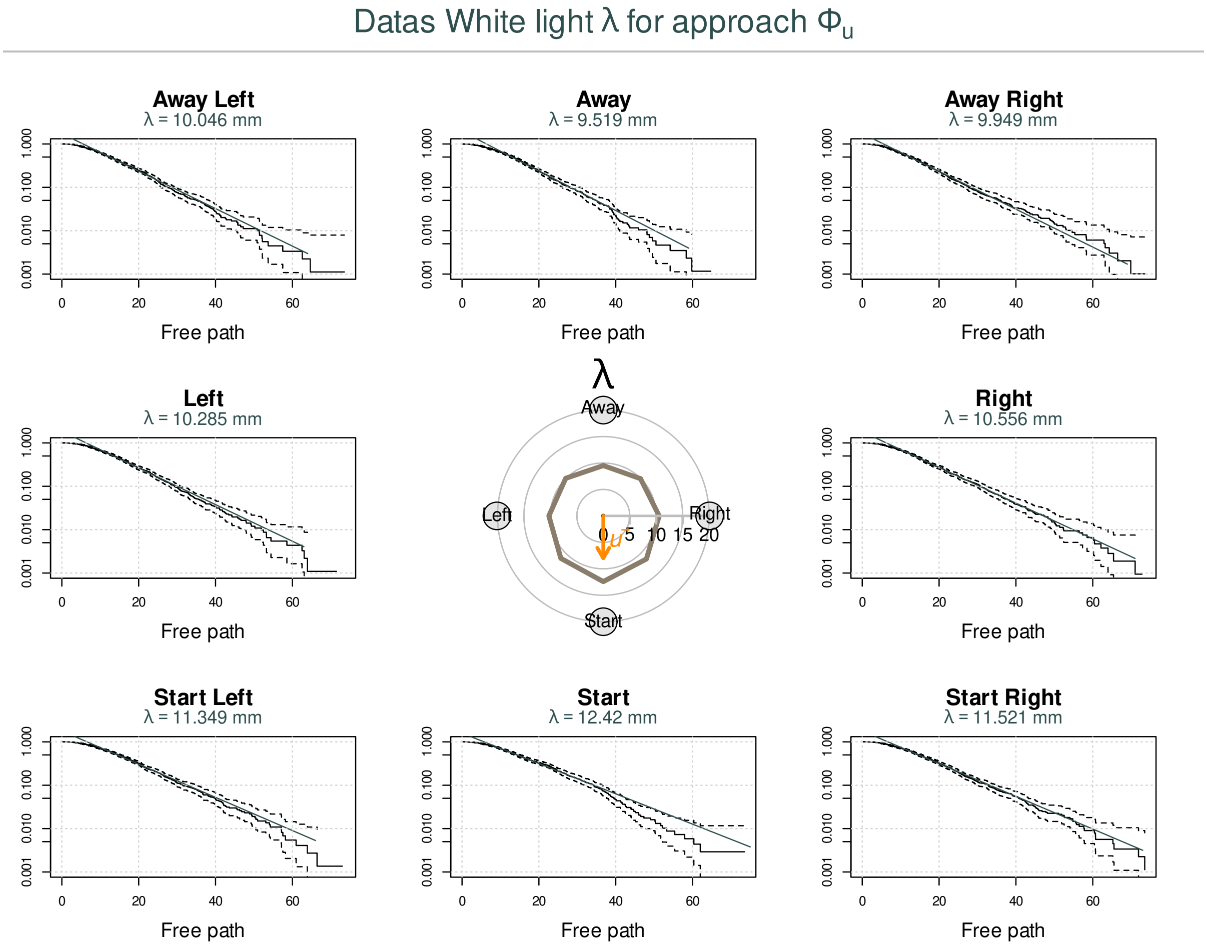

Fig S1.9. White light data: Survival curves (solid curve, the dotted lines indicate a 95% confidence band, log-linear scale) of free paths l as a function of ant orientation with respect to $\vec{u}$ direction pointing back to the starting point ($\Phi_{\vec{u}}$-approach). The straight lines show the weighted linear regressions (fitted to the survival curve values > 10mm) to estimate the slope (its absolute value is the inverse of the mean free path λ). We detect the same tendencies as in Khuong *et al*'s data in Fig. S1.4, but they are not statistically significant on the configuration-wide level (Table S1.3).

Datas Red light λ for approach Φ_u

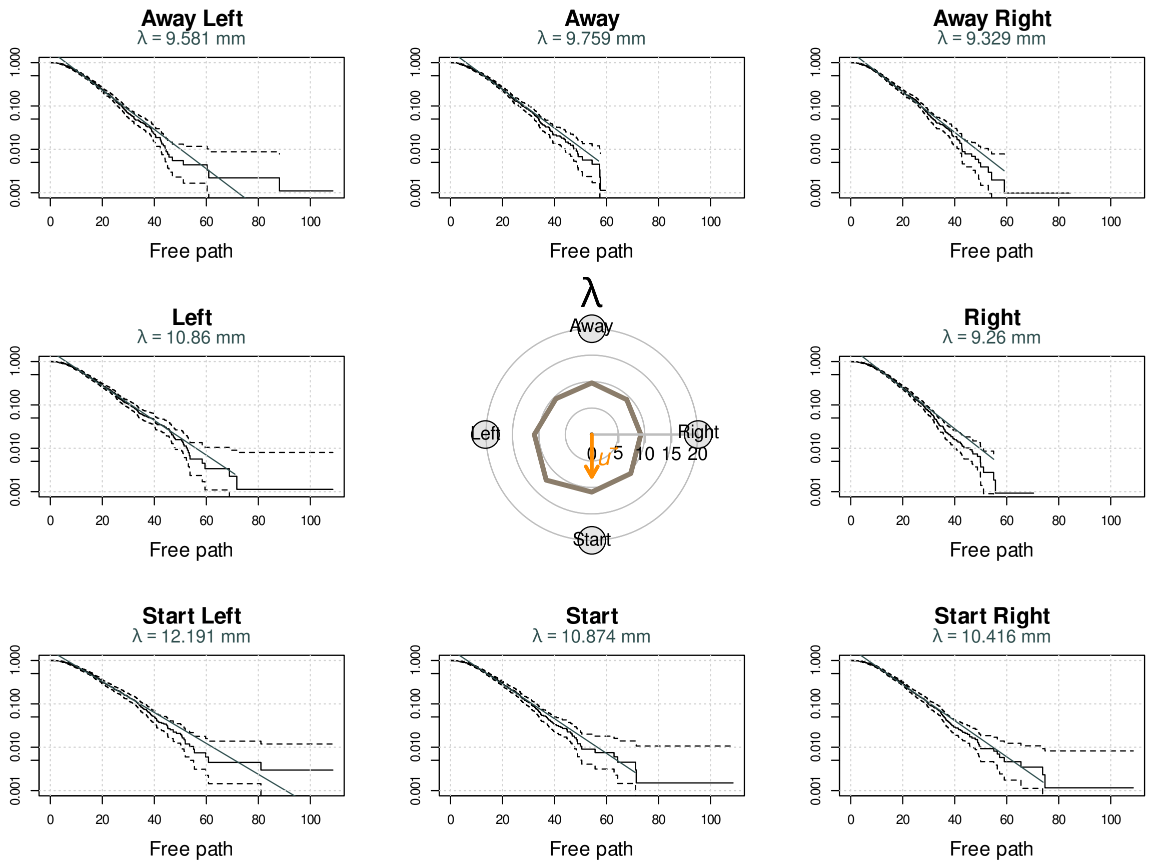

Fig S1.10. Red light data: Survival curves of free paths l as a function of ant orientation with respect to the $\vec{u}$ direction pointing back to the starting point ($\Phi_{\vec{u}}$-approach), organized as in Fig. S1.3. The straight lines show the weighted linear regressions (fitted to the survival curve values > 10mm) to estimate the slope (its absolute value is the inverse of the mean free path λ). The central Figure is the same as Fig. 3e, showing mean free path λ as a function of ant orientation. No directional biases were detected.

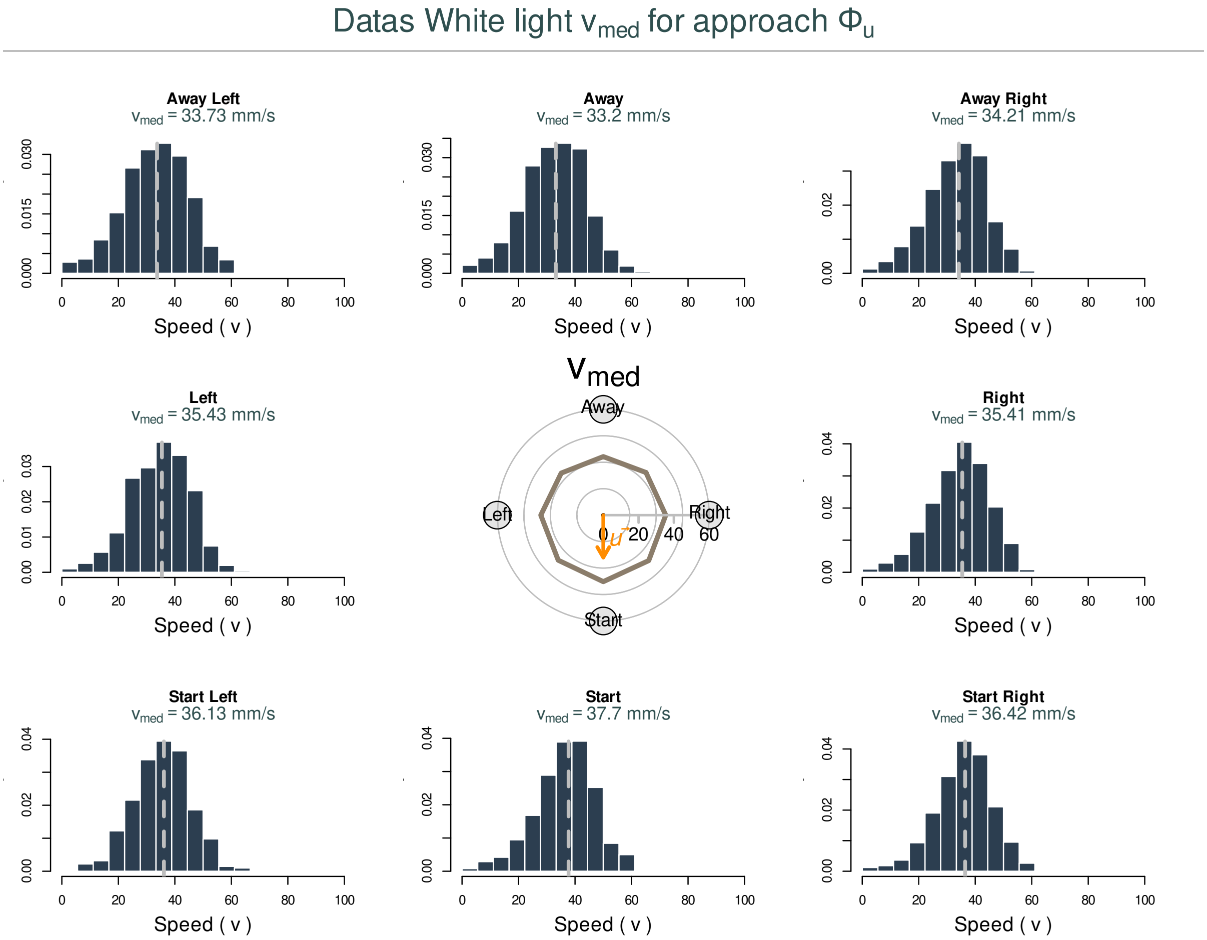

Fig S1.11. White light data: Empirical ant speed v (computed for each free path l) distributions as a function of ant orientation with respect to the camera's x-axis (Φ_x-approach), organized as in Fig. S1.6 (the central Figure is the same as Fig. 3c, showing median speed v_{med} as a function of ant orientation). We detect a clear tendency to move faster when returning to the starting point (compare to Fig. S1.6).

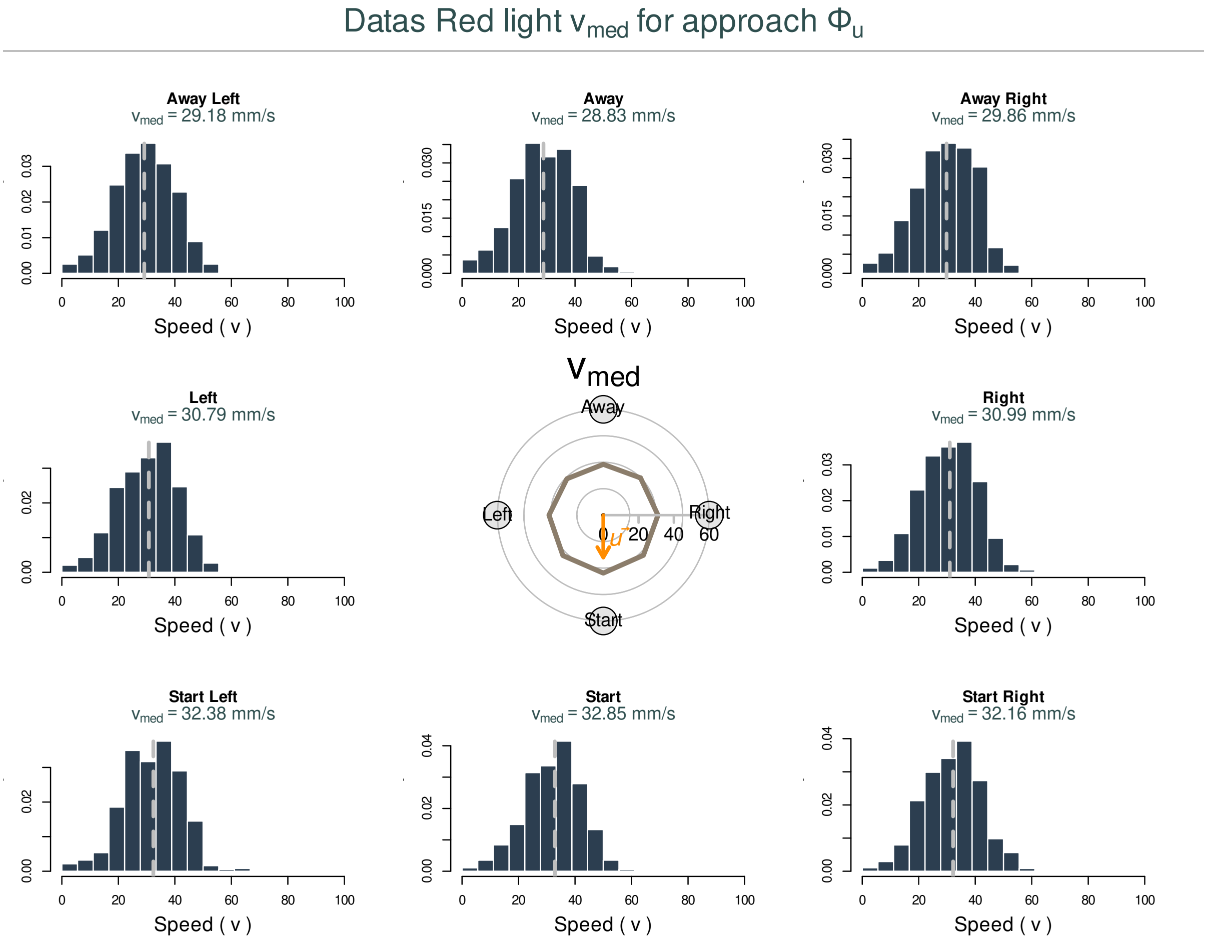

Fig S1.12. Red light data: Empirical ant speed v (computed for each free path l) distributions as a function of ant orientation sector with respect to the $\vec{u}$ direction pointing back towards the starting point ($\Phi_{\vec{u}}$-approach), organized as in Fig. S1.6 (the central Figure is the same as Fig. 3f, showing median speed v_{med} as a function of ant orientation). Ant speed has similar distributions in all orientations.

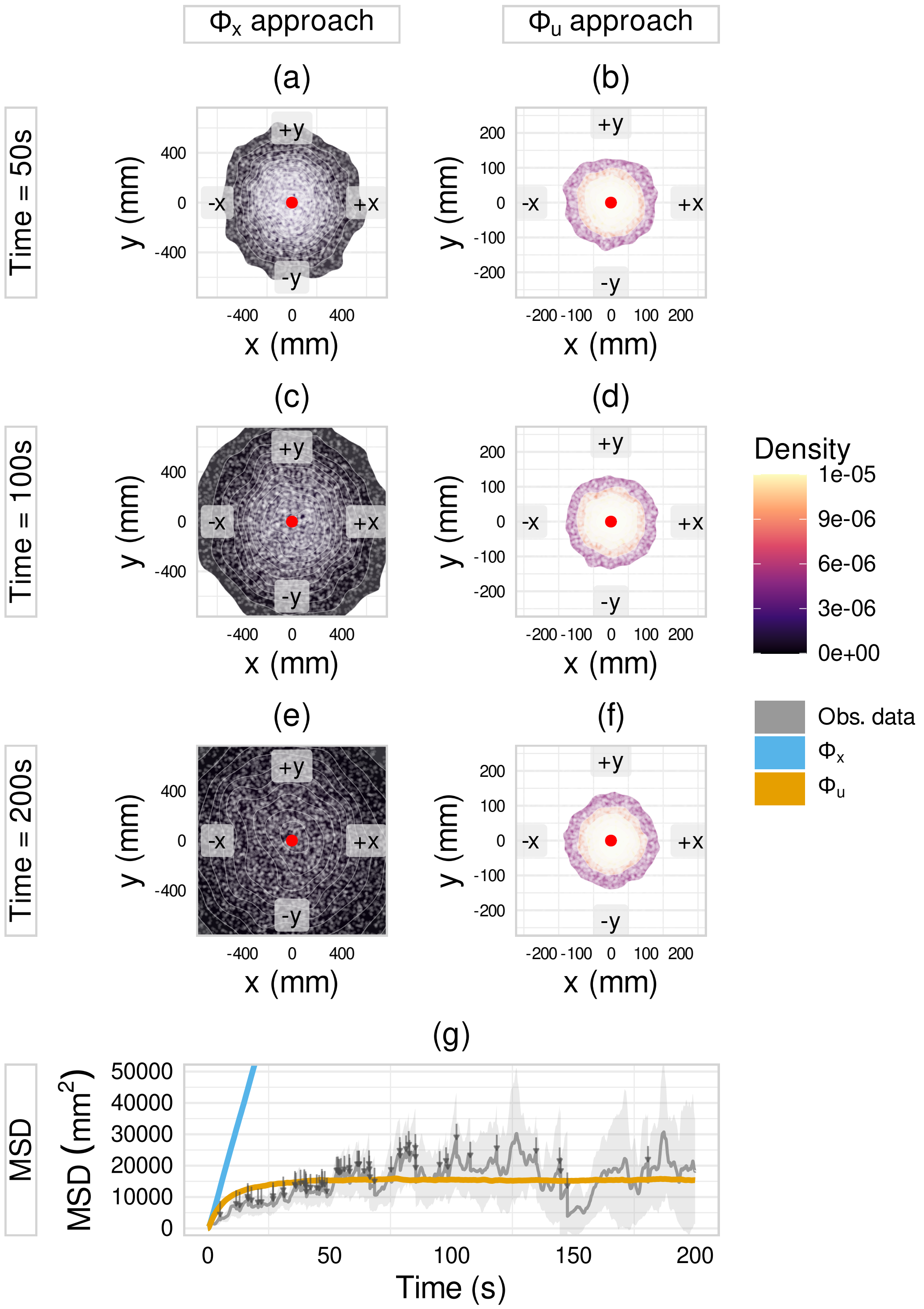

Fig S1.13. Non-parametric simulation of ant dispersal using the reconstructed empirical distributions (Khuong data, Figs. S1.1-S1.6) of ω, l and v in the Φ_x approach (left) and in the $\Phi_{\vec{u}}$ approach (right). The top three lines show density plots at different times: 50s (top line), 100s (second line) and 200s (third line). Note that the spatial scale is not the same between Φ_x and $\Phi_{\vec{u}}$. The bottom line shows experimental mean squared displacement (MSD, mean and 95% confidence band - an arrow indicates the time when a trajectory hits the arena border and no longer contributes to MSD computation) compared to the predicted MSD (blue line for the Φ_x approach, orange line for the $\Phi_{\vec{u}}$ approach).

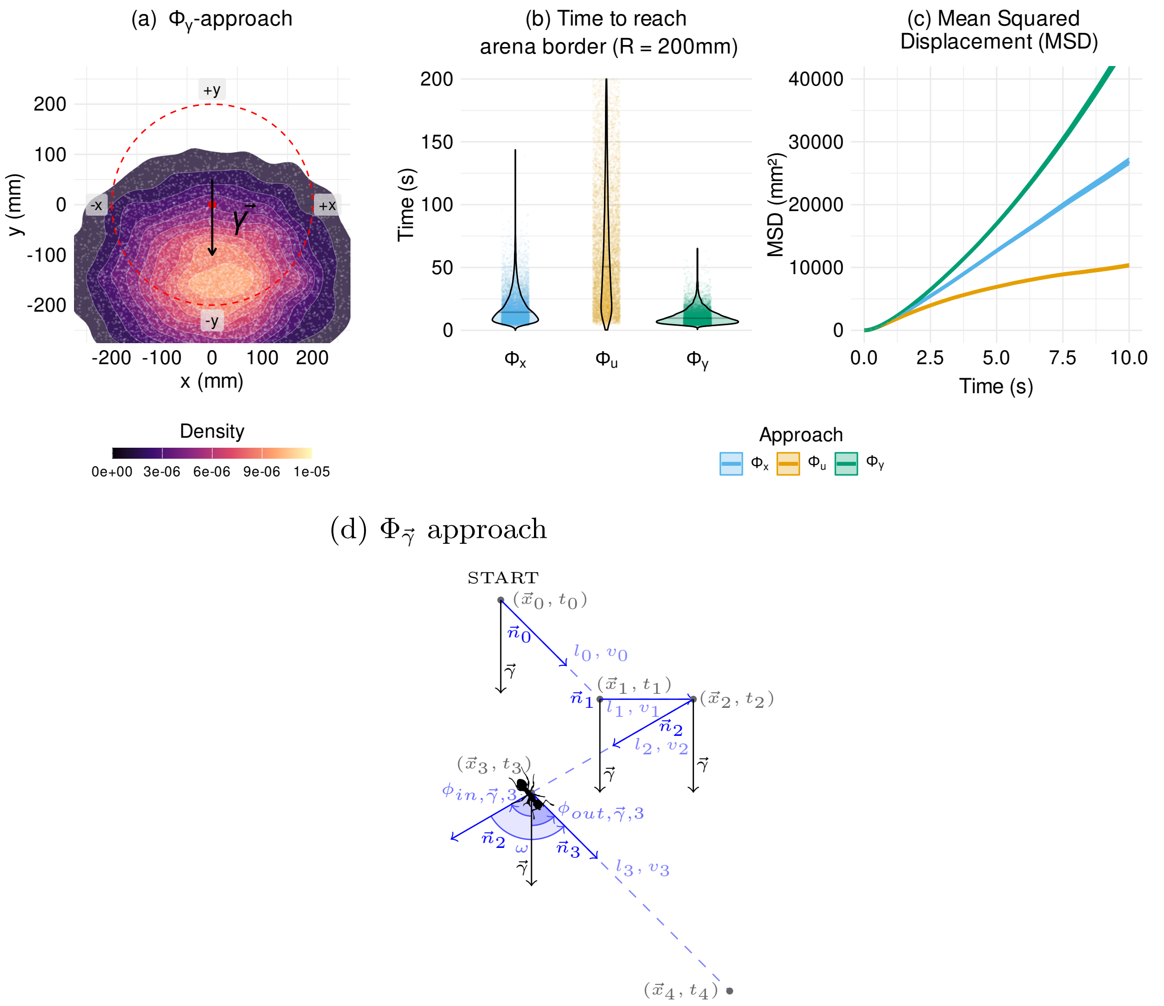

Fig S1.14. Non-parametric simulation of ant dispersal using the reconstructed empirical distributions of ω, l and v in the $\Phi_{\vec{u}}$ approach (as in Figs. S1.2, S1.4, S1.6) but applied to the case where the reference direction $\vec{\gamma}$ always points in the $-y$ axis direction (simulating the case where the ants are far away from their target position, $\Phi_{\vec{\gamma}}$ approach). (a) spatial distribution of ants, placed initially at coordinates $(0, 0)$, after a 10s random walk; (b) violin plots of the times to perform a net displacement of 200mm (red circle in a) in comparison to these times in the Φ_x and $\Phi_{\vec{u}}$ approach (see Fig. 4 in the main text); (c) net squared displacement of simulated random walks in the three approaches. Color code used in (b,c): Φ_x blue, $\Phi_{\vec{u}}$ orange, $\Phi_{\vec{\gamma}}$ green. (d) in the $\Phi_{\vec{\gamma}}$ approach ant heading is characterised with respect to a vector $\vec{\gamma}$ that always points in the same direction (to a far away target position). The notations are as in main Fig. 1.

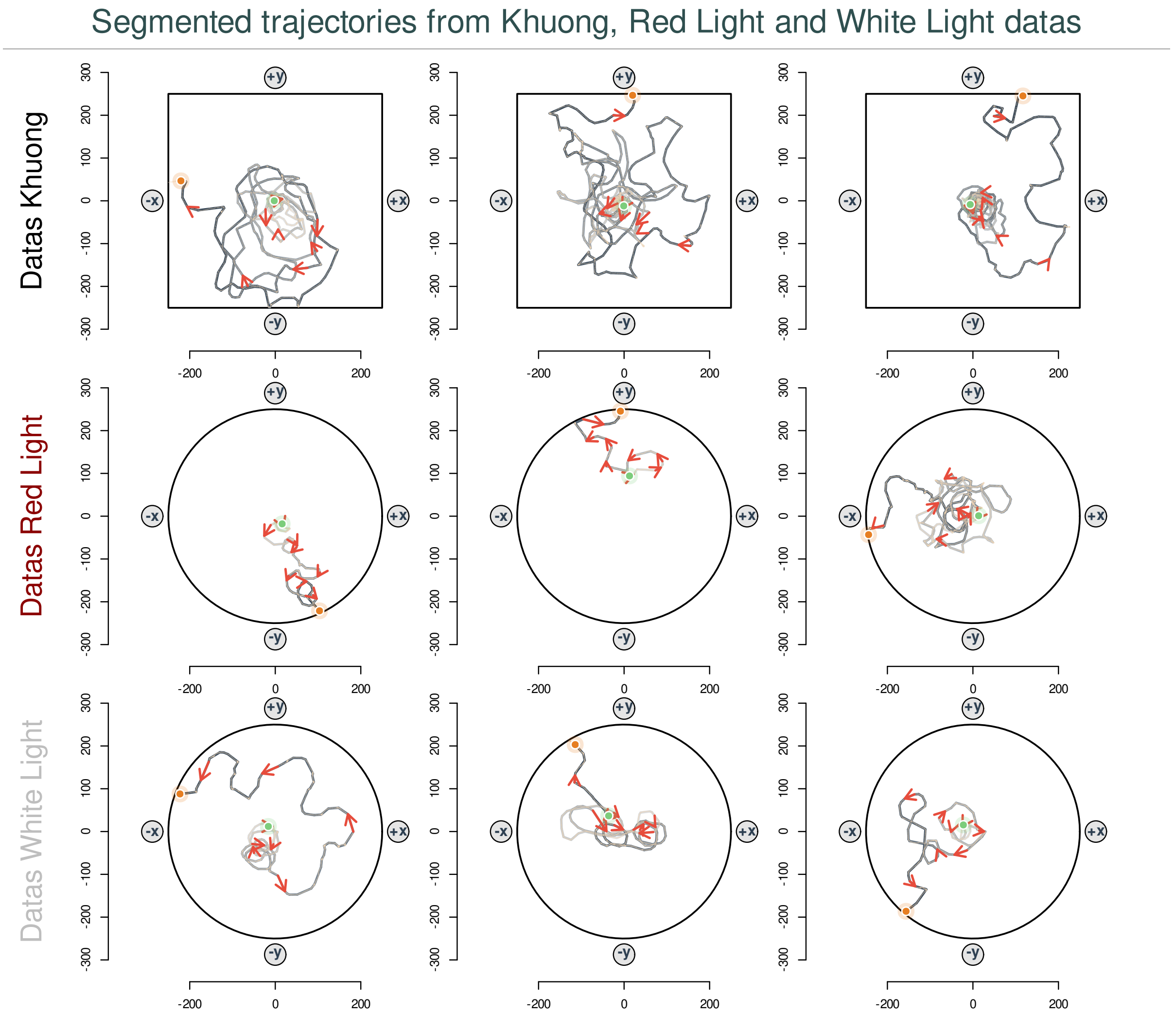

Fig S1.15. Example trajectories for each of the three analyzed data-sets: each plot shows one segmented trajectory (original coordinates added as dots) and the red arrows indicate walking direction. The gray scale of the trajectory refers to time, light gray for the beginning, dark grey for the end. (top line) three trajectories from Khuong *et al* 2013, square canvas; (middle line) three trajectories from the experiments under red light, circular arena; (bottom line) three trajectories from the experiments under white light, circular arena.

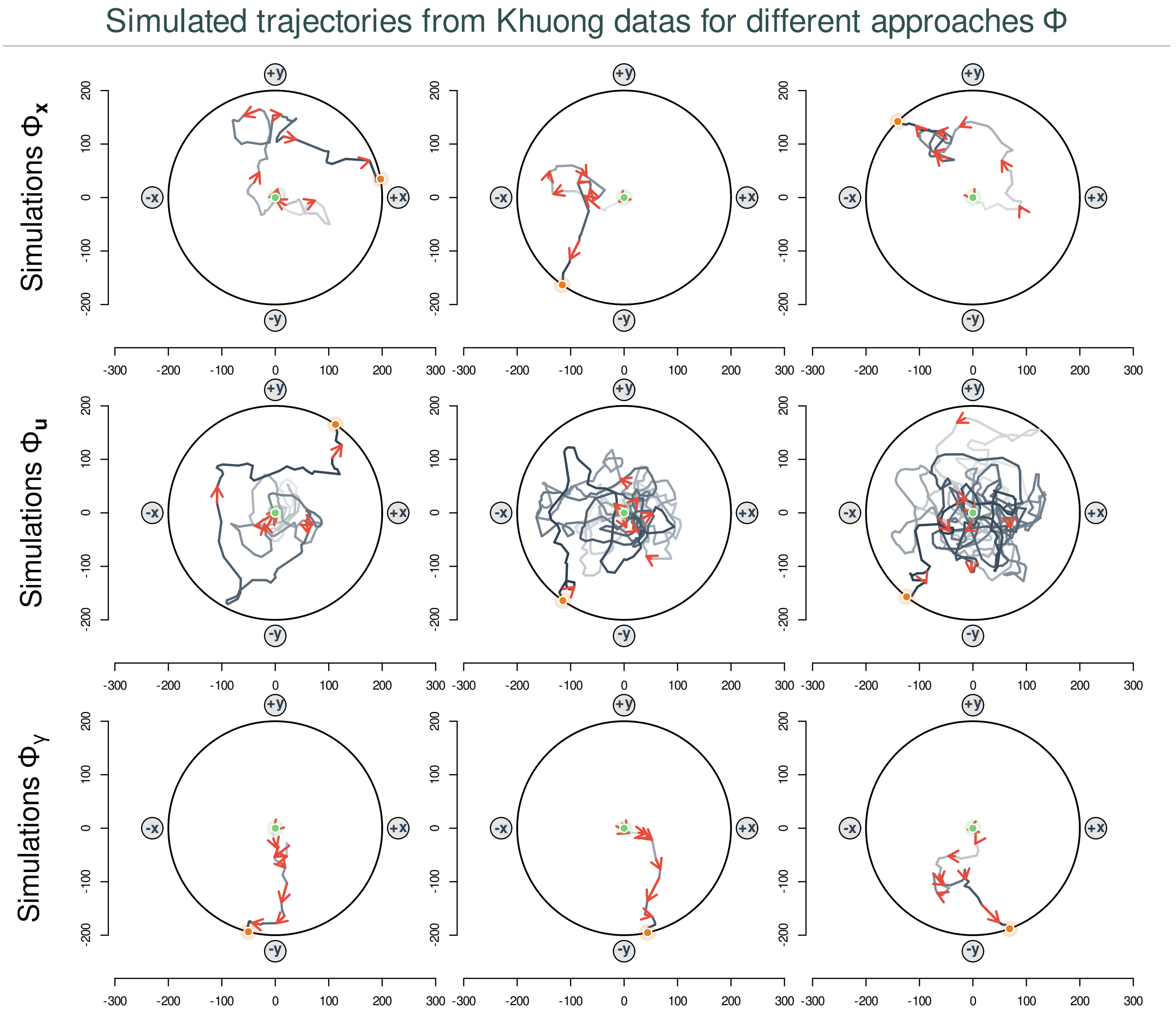

Fig S1.16. Examples of non-parametrically simulated trajectories based on the empirical distributions of the Boltzmann walker variables for the data from Khuong *et al* 2013: (top line) three trajectories with the Φ_x approach (see Figs. S1.1, S1.3 and S1.5); (middle line) with the $\Phi_{\vec{u}}$ approach (see Figs. S1.2, S1.4 and S1.6); (bottom line) with the $\Phi_{\vec{\gamma}}$ approach (see Fig. S1.14, empirical distributions from Figs. S1.2, S1.4 and S1.6).

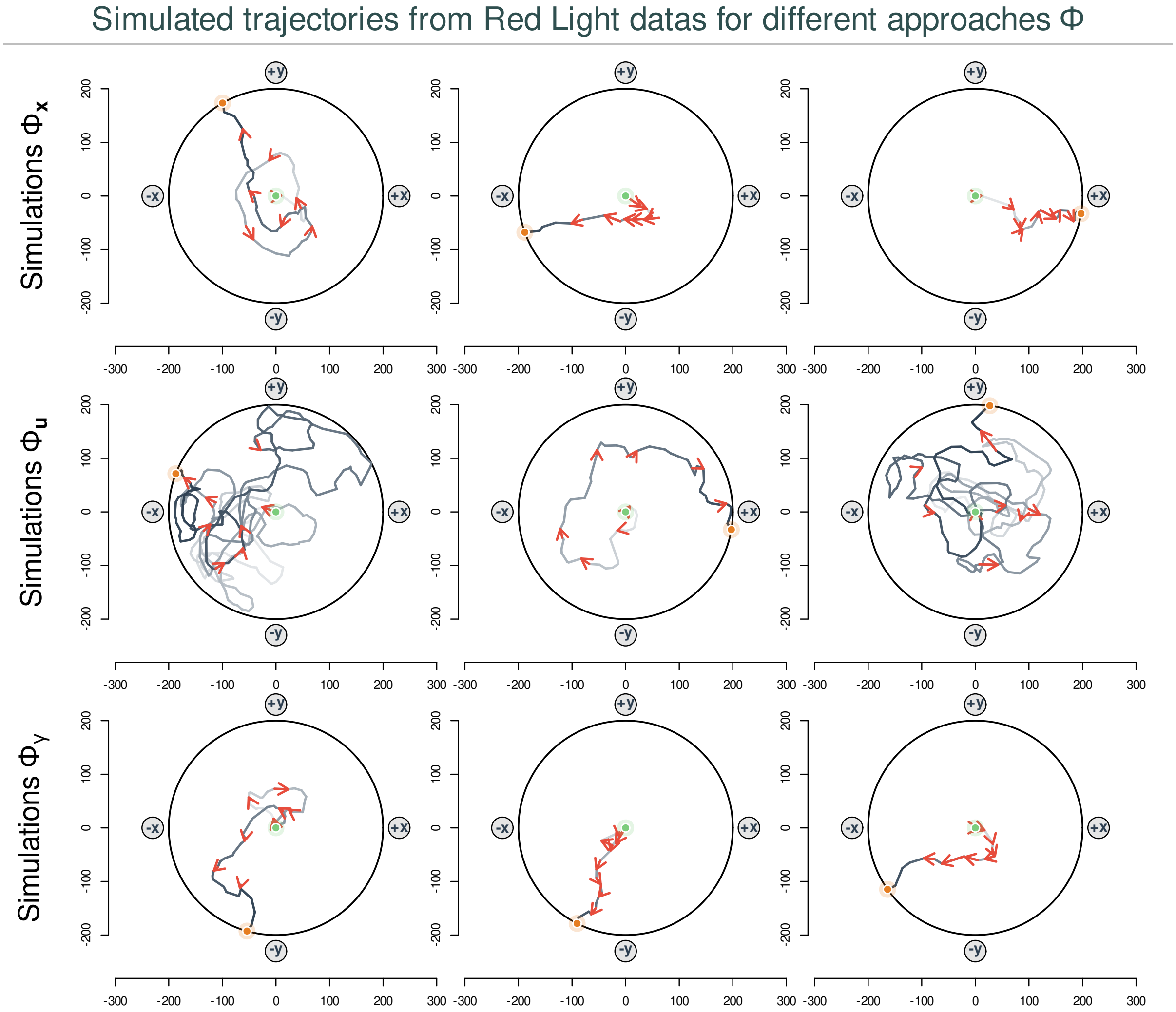

Fig S1.17. Examples of non-parametrically simulated trajectories based on the empirical distributions of the Boltzmann walker variables for the red light data: (top line) three trajectories with the Φ_x approach (empirical distributions not shown); (middle line) three trajectories with the $\Phi_{\vec{u}}$ approach (see Figs. S1.8, S1.10, S1.12); (bottom line) three trajectories with the $\Phi_{\vec{\gamma}}$ approach (as in Fig. S1.14, empirical distributions from Figs. S1.8, S1.10 and S1.12).

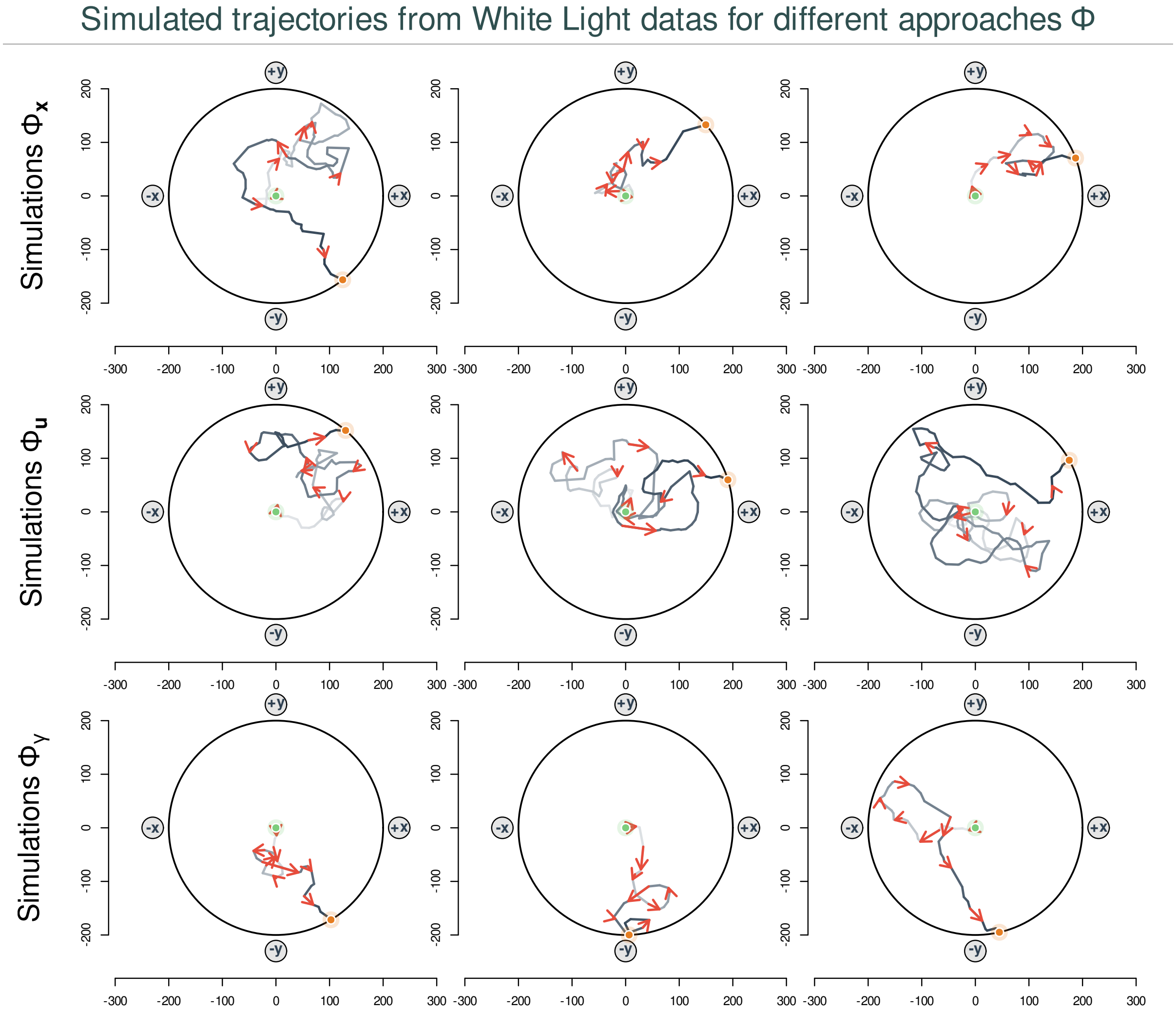

Fig S1.18. Examples of non-parametrically simulated trajectories based on the empirical distributions of the Boltzmann walker variables for the white light data: (top line) three trajectories with the Φ_x approach (empirical distributions not shown); (middle line) three trajectories with the $\Phi_{\vec{u}}$ approach (see Figs. S1.7, S1.9 and S1.11); (bottom line) three trajectories with the $\Phi_{\vec{\gamma}}$ approach (as in Fig. S1.14, empirical distributions from Figs. S1.7, S1.9 and S1.11).

Table S1.1. Detailed statistical results of the linear mixed model comparison between the **Away (or +y)** direction (3 sectors combined) and the **Start (or -y)** direction (3 sectors combined) for parameters v_{med}, g_ω and s_ω. Each line reports the F values, the degrees of freedom, the p values, the mean values M (plus 95%CI) of the **Away (or y)** direction, the **Start (or -y)** direction, and the Effect size (mean difference between the two directions). Statistically significant results (with Bonferroni correction, threshold 0.006) are in bold letters.

Config.	Par.	F	df_{num}	df_{denom}	p	Away		Start		Effect size	
						M	95% CI	M	95% CI	M	95% CI
Khuong Φ_x	v_{med}	2.05	1.00	340.03	.153	52.37	[48.93, 55.80]	53.17	[49.74, 56.61]	-0.81	[-1.92, 0.30]
Khuong Φ_x	g_ω	3.71	1.00	338.40	.055	0.58	[0.56, 0.60]	0.61	[0.58, 0.63]	-0.03	[-0.05, 0.00]
Khuong Φ_x	s_ω	5.42	1.00	336.43	.021	0.01	[-0.02, 0.04]	0.04	[0.02, 0.07]	-0.04	[-0.07, -0.01]
Khuong $\Phi_{\vec{u}}$	v_{med}	3.84	1.00	340.21	.051	51.86	[48.46, 55.26]	53.08	[49.67, 56.48]	-1.22	[-2.44, 0.00]
Khuong $\Phi_{\vec{u}}$	g_ω	78.62	1.00	336.54	< **.001**	0.53	[0.51, 0.56]	0.67	[0.65, 0.70]	**-0.14**	**[-0.17, -0.11]**
Khuong $\Phi_{\vec{u}}$	s_ω	0.12	1.00	330.96	.730	0.02	[-0.02, 0.05]	0.01	[-0.02, 0.05]	0.01	[-0.04, 0.05]
White $\Phi_{\vec{u}}$	v_{med}	34.17	1.00	271.58	< **.001**	34.64	[33.12, 36.17]	36.91	[35.36, 38.46]	**-2.27**	**[-3.03, -1.50]**
White $\Phi_{\vec{u}}$	g_ω	3.64	1.00	272.20	.057	0.72	[0.69, 0.75]	0.75	[0.72, 0.78]	-0.03	[-0.06, 0.00]
White $\Phi_{\vec{u}}$	s_ω	0.69	1.00	273.45	.407	-0.01	[-0.05, 0.03]	0.01	[-0.04, 0.05]	-0.02	[-0.07, 0.03]
Red $\Phi_{\vec{u}}$	v_{med}	16.42	1.00	279.67	< **.001**	30.56	[28.63, 32.48]	32.26	[30.32, 34.20]	**-1.70**	**[-2.53, -0.88]**
Red $\Phi_{\vec{u}}$	g_ω	1.03	1.00	279.23	.310	0.68	[0.65, 0.71]	0.69	[0.66, 0.72]	-0.02	[-0.05, 0.02]
Red $\Phi_{\vec{u}}$	s_ω	1.14	1.00	279.97	.286	-0.03	[-0.07, 0.02]	0.00	[-0.04, 0.05]	-0.03	[-0.09, 0.03]

Table S1.2. Detailed statistical results of the linear mixed model comparison between the **Left (or -x)** direction (3 sectors combined) and the **Right (or +x)** direction (3 sectors combined) for parameters v_{med}, g_ω and s_ω. Each line reports the F values, the degrees of freedom, the p values, the mean values M (plus 95%CI) of the **Left (or -x)** direction, the **Right (or +x)** direction, and the Effect size (mean difference between the two directions). Statistically significant results (with Bonferroni correction, threshold 0.006) are in bold letters.

Config.	Par.	F	df_{num}	df_{denom}	p	Left		Right		Effect size	
						M	95% CI	M	95% CI	M	95% CI
Khuong Φ_x	v_{med}	0.80	1.00	203.95	.371	52.08	[48.63, 55.52]	52.66	[49.21, 56.11]	0.58	[-0.70, 1.87]
Khuong Φ_x	g_ω	1.59	1.00	199.83	.209	0.58	[0.55, 0.60]	0.60	[0.57, 0.62]	0.02	[-0.01, 0.05]
Khuong Φ_x	s_ω	0.15	1.00	203.81	.700	0.03	[0.00, 0.06]	0.04	[0.01, 0.07]	0.01	[-0.03, 0.05]
Khuong $\Phi_{\vec{u}}$	v_{med}	5.69	1.00	204.12	.018	54.05	[50.55, 57.56]	52.23	[48.72, 55.74]	-1.83	[-3.33, -0.32]
Khuong $\Phi_{\vec{u}}$	g_ω	0.07	1.00	203.38	.793	0.62	[0.59, 0.65]	0.61	[0.58, 0.64]	-0.01	[-0.05, 0.03]
Khuong $\Phi_{\vec{u}}$	s_ω	67.40	1.00	193.10	$<$ **.001**	0.11	[0.07, 0.14]	-0.09	[-0.12, -0.05]	**-0.19**	**[-0.24, -0.15]**
White $\Phi_{\vec{u}}$	v_{med}	0.02	1.00	159.74	.900	35.54	[34.00, 37.08]	35.60	[34.07, 37.14]	0.06	[-0.90, 1.02]
White $\Phi_{\vec{u}}$	g_ω	0.63	1.00	161.78	.427	0.73	[0.70, 0.76]	0.75	[0.72, 0.78]	0.01	[-0.02, 0.05]
White $\Phi_{\vec{u}}$	s_ω	39.81	1.00	158.19	$<$ **.001**	0.07	[0.02, 0.11]	-0.09	[-0.14, -0.05]	**-0.16**	**[-0.21, -0.11]**
Red $\Phi_{\vec{u}}$	v_{med}	0.60	1.00	165.96	.439	30.97	[29.01, 32.93]	31.40	[29.44, 33.36]	0.43	[-0.67, 1.53]
Red $\Phi_{\vec{u}}$	g_ω	0.06	1.00	164.58	.803	0.69	[0.65, 0.72]	0.69	[0.66, 0.73]	0.01	[-0.04, 0.05]
Red $\Phi_{\vec{u}}$	s_ω	3.32	1.00	161.65	.070	0.03	[-0.03, 0.09]	-0.04	[-0.10, 0.02]	-0.07	[-0.14, 0.01]

Table S1.3. Detailed statistical results of the mixed effect Cox model comparison of mean free path λ between the **Left (or -x)** direction (3 sectors combined) and the **Right (or +x)** direction (3 sectors combined, or the **Away (or +y)** direction (3 sectors combined) and the **Start (or -y)** direction (3 sectors combined). Each line reports the z statistic, the p value and the Effect size or odds ratio (length ratio between the two directions). Statistically significant results (with Bonferroni correction, threshold 0.006) are in bold letters.

Config.	Direction	z	p	Odd's ratio	95% CI
Khuong Φ_x	$+y$ *vs.* $-y$	-0.06	.953	0.99	[0.81, 1.23]
Khuong Φ_x	$-x$ *vs.* $+x$	1.60	.109	1.23	[0.95, 1.59]
Khuong $\Phi_{\vec{u}}$	Away *vs.* Start	-6.91	$<$ **.001**	**0.46**	**[0.37, 0.58]**
Khuong $\Phi_{\vec{u}}$	Left *vs.* Right	-0.48	.632	0.94	[0.73, 1.21]
White light $\Phi_{\vec{u}}$	Away *vs.* Start	-2.03	.042	0.78	[0.62, 0.99]
White light $\Phi_{\vec{u}}$	Left *vs.* Right	-0.38	.707	0.94	[0.70, 1.28]
Red light $\Phi_{\vec{u}}$	Away *vs.* Start	-1.39	.166	0.85	[0.67, 1.07]
Red light $\Phi_{\vec{u}}$	Left *vs.* Right	-0.81	.418	0.89	[0.67, 1.18]
